# Selective HDAC6 inhibitors improve anti-PD-1 immune checkpoint blockade therapy by decreasing the anti-inflammatory phenotype of macrophages and down-regulation of immunosuppressive proteins in tumor cells

**DOI:** 10.1038/s41598-019-42237-3

**Published:** 2019-04-16

**Authors:** Tessa Knox, Eva Sahakian, Debarati Banik, Melissa Hadley, Erica Palmer, Satish Noonepalle, Jennifer Kim, John Powers, Maria Gracia-Hernandez, Vasco Oliveira, Fengdong Cheng, Jie Chen, Cyril Barinka, Javier Pinilla-Ibarz, Norman H. Lee, Alan Kozikowski, Alejandro Villagra

**Affiliations:** 1 The George Washington University, Washington, DC USA; 2 H. Lee Moffitt, Tampa, FL USA; 3 Hospital Episcopal San Lucas, Ponce, PR USA; 4 Institute of Biotechnology of the Czech Academy of Sciences, BIOCEV, Vestec, Czech Republic; 5 StarWise LLC, Madison, WI USA

**Keywords:** Skin cancer, Tumour immunology

## Abstract

Histone deacetylases (HDACs) are involved in diverse cellular regulatory mechanisms including non-canonical functions outside the chromatin environment. Several publications have demonstrated that selective HDAC inhibitors (HDACi) can influence tumor immunogenicity and the functional activity of specific immune cells. In particular, the selective inhibition of HDAC6 has been reported to decrease tumor growth in several malignancies. However, there is still no clarity about the cellular components mediating this effect. In this study, we evaluated the HDAC6i Nexturastat A as a priming agent to facilitate the transition of the tumor microenvironment from “cold” to “hot”, and potentially augment immune check-point blockade therapies. This combination modality demonstrated to significantly reduce tumor growth in syngeneic melanoma tumor models. Additionally, we observed a complete neutralization of the up-regulation of PD-L1 and other immunosuppressive pathways induced by the treatment with anti-PD-1 blockade. This combination also showed profound changes in the tumor microenvironment such as enhanced infiltration of immune cells, increased central and effector T cell memory, and a significant reduction of pro-tumorigenic M2 macrophages. The evaluation of individual components of the tumor microenvironment suggested that the *in vivo* anti-tumor activity of HDAC6i is mediated by its effect on tumor cells and tumor-associated macrophages, and not directly over T cells. Overall, our results indicate that selective HDAC6i could be used as immunological priming agents to sensitize immunologically “cold” tumors and subsequently improve ongoing immune check-point blockade therapies.

## Introduction

Most standard therapies for cancer patients focus primarily on surgery, radiation and targeted chemotherapies. Unfortunately, patients are often refractory to treatment or experience relapse. In addition, the side effects that result from these therapies have a major impact on the quality of life in patients, which can considerably limit the use of these therapies. Most recently, the development and clinical use of immune-based therapies such as monoclonal antibodies that work by blocking immunosuppressive signaling pathways, have revolutionized the treatment of several cancer types, including melanoma^1^.

Collectively, the clinical data obtained thus far suggest that patients’ response to treatment with the immune checkpoint blockade anti-PD-1 varies broadly among different cancers^2^. In melanoma, a recent study found that the anti-PD-1 antibody nivolumab produced objective responses (OR) in 44% of patients^3^. It is important to emphasize the identification of a potential correlation between the observed objective response and PD-L1 expression. While an OR of 54% was achieved within the PD-L1 positive patient population (≥1% PD-L1), the OR in PD-L1-negative patients (<1% PD-L1) was 35%. One plausible explanation proposes that PD-1 blockade enhances T-cell function and the subsequent production of interferon-gamma (IFNγ) and other pro-inflammatory cytokines. These cytokines, in turn, have been described as powerful enhancers of immunosuppressive mediators in tumor cells, including PD-L1, PD-L2, and galectin-9. Thus, the high levels of PD-L1 observed in patients under immune blockade treatment, might be a direct consequence of a higher exposition to pro-inflammatory cytokines^4^. There remains a critical lack of knowledge about the regulatory mechanisms controlling the expression of other immunosuppressive pathways, particularly by pro-inflammatory cytokines. It has been proposed that PD-L2, which is mainly expressed by professional antigen presenting cells (APCs), does not bind exclusively to PD-1, as demonstrated by its ability to interfere with T-cell function even in PD-1 knockout mice^5^. This opens the possibility that a pro-inflammatory tumor microenvironment triggered by the PD-1 blockade could induce negative feedback to activate other immunosuppressive pathways in different immune cells. Consequently, as tumors evolve under intense immune pressure, they develop mechanisms that lessen their immune visibility, thereby evading further immunological assaults. Therefore, the challenge to identify the most potent treatment combinations to maximize therapeutic benefits, by increasing immunogenicity and minimizing immune-related adverse events (irAEs), has become a major goal in cancer research. This active search for new therapeutic combinations has identified numerous potential molecular targets. Among them, there is emerging interest in the understanding of the role of histone deacetylases (HDACs) in the control of immuno-modulatory pathways, especially those directly involved in the regulation of immune check-point modulators.

Initially, histone deacetylases (HDACs) were characterized as enzymes that remove acetyl groups from histones, establishing a silent chromatin structure. However, HDACs have recently been shown as acting over a wider spectrum of substrate proteins, involved in a range of cellular processes that extend beyond the chromatin environment including regulatory functions that vary with their tissue expression, cellular compartmentalization, and stage of cellular differentiation^6^. There are a total of eighteen HDACs that have been identified and are divided into four classes^7^: Class I includes HDAC1, 2, 3 and 8; Class II includes HDAC4, 5, 6, 7, 9 and 10; Class III consists of members of the sirtuin HDACs; and Class IV includes the most recently discovered HDAC, HDAC11^7^. There are non-histone proteins, including transcription factors, that can also be regulated by HDACs^6^. As a result of their influence on chromatin structure and transcription factors, as well as their involvement in multiple other cellular processes, HDACs are attractive molecular targets to control gene expression and the function of specific proteins^6^.

Thus far, various non-selective HDACi (pan-HDACi) have shown significant antitumor activity in preclinical and clinical models. While a promising therapeutic option for hematologic cancer patients, the results of pan-HDACi use are inconsistent in solid tumors. These mixed effects are likely because these drugs are non-specific and target more than one of the eighteen known HDAC isoforms which translates in multiple non-uniform effects on immune and survival-related cellular pathways. The toxic side effects observed in patients have included fatigue, severe gastrointestinal distress, hematological side effects and major cardiotoxicity^8^. Collectively, these adverse events diminish the enthusiasm for the use of pan-HDACi as either single or combination therapy in the context of anti-cancer drug treatment programs. As a result, the approval of these drugs by the FDA is limited to a reduced number of cancer types.

The use of specific inhibitors for individual HDACs may diminish the observed toxicity induced by pan-HDACi while maintaining specificity for the desired therapeutic target and anti-oncogenic outcomes necessary for potential new and effective therapies. Based on this premise, a number of class-specific and isotype-specific HDACi have been shown to have anti-tumorigenic effects. Among them, HDAC6i have gained special attention as possible anti-cancer agents. Recent reports have shown that HDAC6 plays a critical role as an immune checkpoint regulator in primary human melanoma cells^9^. While the use of nonspecific HDACi, such as panobinostat, are known to increase the expression of the immunosuppressive proteins program death ligand-1 (PD-L1) and programmed death ligand-2 (PD-L2) on the cell surface of tumor cells^10^, selective HDAC6i have shown the opposite effect by either blunting or decreasing the expression of PD-L1.

Although the cytotoxic effects of HDAC6i are minimal in isolated *in vitro* cell cultures^11^, these agents can effectively impair tumor growth and progression in murine *in vivo* models without inducing major adverse events; a characteristic highly desirable in the advancement of drug compounds into the clinic, and also clearly differentiating from the prevailing cytotoxic-centric paradigm previously assigned to HDACi. Moreover, several reports have shown that HDAC6 expression and function is altered in other non-cancer related conditions^12^. HDAC6 is known to be overexpressed in many cancer types and the complete genetic abrogation of HDAC6 does not impair normal cellular functions^13^.

Here, we report that the combination therapy of anti-PD-1 blocking antibodies with selective HDAC6i significantly decreases tumor growth compared to each agent alone. Additionally, we identified an increased infiltration of CD8 and natural killers (NK) cells, and a diminished presence of pro-tumoral M2 macrophages in the tumor microenvironment (TME) associated with HDAC6 treatment. All the above were accompanied by an important overall change in the cytokine milieu favoring a pro-inflammatory ‘hot” TME. Collectively, these data provide the initial rationale to design new anti-PD-1 and HDAC6i combination therapies for clinical trials in melanoma and other solid tumors.

## Results

### The up-regulation of PD-L1 in anti-PD-1 treated mice is mediated by IFNγ

**Figure 1:**
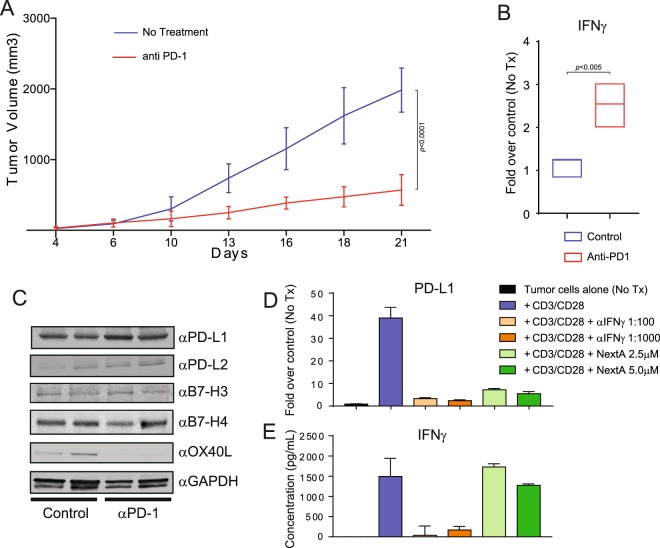
The up-regulation of PD-L1 in anti-PD-1 treated mice is mediated by IFNγ. (**A**) C57BL/6 mice were subcutaneously injected with 1 × 10^6^ SM1 murine melanoma tumor cells. Mice were treated with 15 mg/kg anti-PD-1 or a vehicle control for 21 days. Tumor nodules were isolated to evaluate the expression of IFNγ by qRT-PCR (**B**), and PD-L1, PD-L2, B7-H3, B7-H4, OX40L, and GAPDH by immunoblot (**C**). SM1 melanoma cells were treated *in vitro* with NextA or vehicle and then co-cultured with CD3/CD28 activated splenocytes in the presence or absence of IFNγ blocking antibody at 1:1000 and 1:100 dilutions. Then, the expression of PD-L1 was analyzed by qRT-PCR (**D**), and the expression of IFNγ by ELISA (**E**).

The overexpression of PD-L1 on tumor cells is widely accepted as an adaptive resistance mechanism to facilitate tumor survival and cancer immune evasion through the inhibition of cytotoxic T cell function^14^. Despite this, recent studies have shown that elevated expression of PD-L1 in tumors correlates with better response rate (RR), progression-free survival (PFS), and overall survival (OS) to anti-PD-1-directed therapy in melanoma and other types of cancer^15^. It has also been proposed that the observed upregulation of PD-L1 on tumor cells could be a direct consequence of IFNγ production by activated tumor-infiltrating T cells, which is associated with a better prognostic outcome^16^. We explored this prospect in mice challenged with murine melanoma SM1 cells, a BRAFV600E mutant tumor model propagated by continuous *in vivo* passaging^17^, and subsequently treated with either anti-PD-1 blocking antibody or vehicle control. As expected, the tumor growth was significantly diminished in the anti-PD-1 arm (Fig. 1A), which was associated with an increase in the presence of secreted IFNγ in the TME when compared to the no treatment group (Fig. 1B). The high levels of IFNγ were also accompanied by increased levels of PD-L1 and PD-L2 in tumor cells (Fig. 1C). Additionally, we observed minimal differences in the expression of B7-H3 and B7-H4, and an important reduction of the expression of OX-40L.

To verify that the up-regulation of PD-L1 in tumor cells is a direct effect of the IFNγ present in the TME, we treated SM1 melanoma cells *in vitro* with NextA or vehicle control and then co-cultured with CD3/CD28 activated splenocytes in the presence of anti-PD1 antibody. IFNγ blocking antibody was added at 1:1000 and 1:100 dilutions. As shown in Fig. 1D, the expression of PD-L1 analyzed by qRT-PCR in SM1 cells was up-regulated when co-cultured with activated T cells. However, this up-regulation was diminished after adding IFNγ blocking antibody at 1:1000 dilution and was completely abrogated at 1:100 dilution, even in the presence of the anti-PD1 antibody. A similar down-regulation of PD-L1 was found when adding NextA to the co-cultured cells (Fig. 1D, last two columns), suggesting that this HDAC6i can neutralize the effect of anti-PD1 antibody mediated upregulation of IFNγ on the production of PD-L1. Importantly, we observed that treatment with NextA did not affect the production of soluble IFNγ (Fig. 1E), indicating that this drug was modulating the IFNγ signaling pathway directly in tumors and not over T cells.

### NextA improves the anti-tumor activity of anti-PD-1 immune checkpoint blockade

HDAC6 is a key regulator of the expression of PD-L1 in tumor cells, and selective HDAC6i impairs the up-regulation of PD-L1 upon cytokine treatment^9^. This regulatory mechanism is mediated by STAT3, which requires functional HDAC6 to be phosphorylated and subsequently translocated to the nucleus to activate the PD-L1 gene. The inhibitory effect of HDAC6i on the production of PD-L1 has also been observed in other immunosuppressive proteins such as PD-L2, B7-H3, and B7-H4 in human primary melanoma biopsies and cell lines^9^. Following these early findings, we asked whether selective HDAC6i affected the expression of immunosuppressive proteins in murine melanoma SM1 cells. In preliminary screenings, we observed that treatment with NextA decreased the IFNγ-mediated up-regulation of PD-L1 and PD-L2, and slightly decreased the expression of B7-H3 and B7-H4 (Fig. Suppl. 1A,B). However, no major differences were observed in the expression of galectin-9, ICOS-L and CD70. Similarly, we observed that the absence of HDAC6 affected the expression of the same proteins from lung tissue isolated from HDAC6 knock-out mice (Fig. Suppl. 1C).

**Figure 2:**
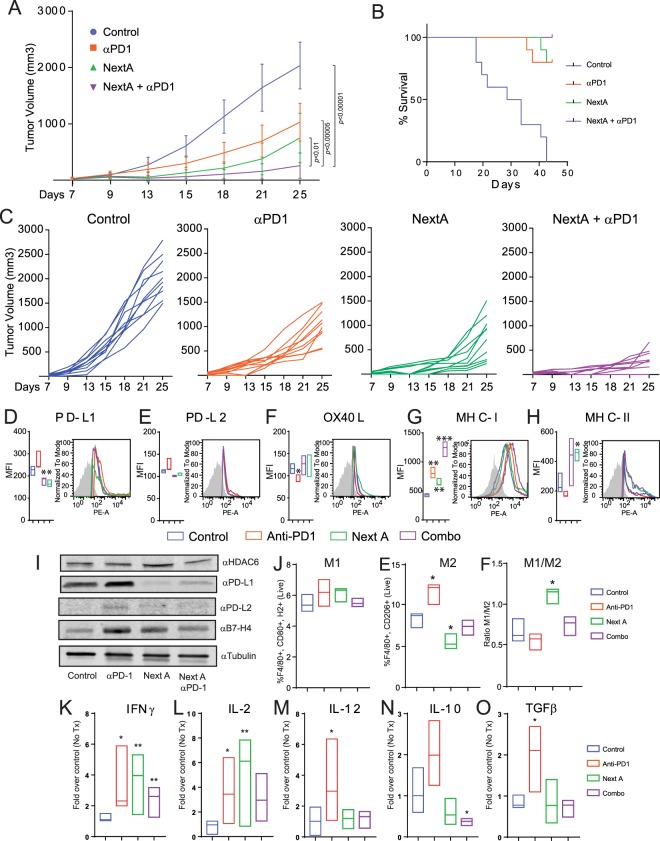
NextA improves the anti-tumor activity of anti-PD-1 immune checkpoint blockade. (**A**) C57BL/6 mice were subcutaneously injected with 1 × 10^6^ SM1 murine melanoma tumor cells. Mice were treated with a vehicle control, 3 mg/kg anti-PD-1, 15 mg/kg NextA, or a combination of both agents for 25 days. (**B**) Kaplan-Meier survival plot of the previous study. (**C**) Individual group plots representation for the previous study. (**D**–**H**) Tumors were collected at the end point from mice treated with a vehicle control, anti-PD-1, NextA, or combination of both agents and the presence of the costimulatory markers PD-L1, PD-L2, OX40L, MHC class I, and MHC class II was evaluated by flow cytometry. (**I**) The expression of PD-L1, PD-L2 and GAPDH was evaluated by immunoblot. (**J**) Tumors were collected at the end point from mice treated with vehicle control, anti-PD-1, NextA, or a combination of both agents and the presence of M1 and M2 surface markers were evaluated by flow cytometry. M1/M2 ratios were calculated from three independent populations from M1 and M2. (**K**–**O**) Total RNA was isolated from the same aforementioned sample conditions and qRT-PCR was done to evaluate the expression of IFNγ, IL-2, IL-12, IL-10 and TGFβ.

In addition to the effect of the abrogation of HDAC6 on the expression of immunosuppressive proteins, it has been shown that the genetic and enzymatic inhibition of HDAC6 induces the expression of MHC class I as well as several tumor- associated antigens including gp100, MART1, TYRP1, and TYRP2^11^. Taking the above into consideration, we hypothesized that the inhibition of HDAC6 could improve the anti-tumor effect of anti-PD-1 blockade by decreasing the expression of molecules involved in immunosuppressive pathways (i.e. PD-L1) and enhancing the antigen presentation machinery. To explore this possibility, we first evaluated the effective dose range for each agent in the combination treatment by performing a dose titration of NextA (Fig. Suppl. 2A), and the anti-PD-1 blocking antibody (Fig. Suppl. 1B), respectively. Since our main goal was to determine whether the combination of these two agents improved the reduction of tumor growth when compared to single agents, we selected the suboptimal concentrations of 15 mg/kg for NextA and 3.0 mg/kg for anti-PD-1. However, we also observed significant differences in tumor growth between groups at other dose concentrations up to 25 mg/kg NextA and 15 mg/kg anti-PD-1. Of note, all treatments in these initial experiments started as soon as the subcutaneous tumors were palpable, usually around 4–7 days post inoculation. The experiment shown in Fig. 2A–C demonstrates that the reduction in tumor growth at day 25 for the combination treatment (257.3 mm^3^) was significantly smaller than those mice that were treated with either NextA (750.1 mm^3^) or anti-PD-1 (1030 mm^3^) in comparison with the control group (2034 mm^3^). Statistically significant (P < 0.0001) differences in tumor growth could be recorded as early as day 13 in the NextA (55.83 mm^3^) and NextA/anti-PD-1 (34.35 mm^3^) groups and at day 15 in the anti-PD-1 group (299.1 mm^3^). The average survival of control mice was 29 days, whereas the mice in each of the treatment groups showed a robust increase in overall survival. By day 44, all mice in the NextA/anti-PD-1 group were still alive (Fig. 2B). In particular, within the depicted experiment, we observed four mice in the combination group that reached total remission of tumor growth; an event not observed in any of the single treatments. This outcome occurred in each experimental repetition with an average of 10–25% in the combination arm. Within the control study arms, mice were treated with either the vehicle control (1X PBS) or IgG2a as an isotype control **(**Fig. Suppl. 1A). After 25 days of tumor growth, there was no statistically significant difference in tumor size between the control groups.

We also performed similar experiments using the B16-F10 murine melanoma cell line. Mice bearing tumors from this cell line also responded to the single and combination arms. However, statistical differences were found only between the combination group and the anti-PD-1 treatment (Fig. Suppl. 3A–C). Despite the minimal differences in tumor growth, the survival rates clearly demonstrated a superior effect of the combination of NextA and anti-PD-1 when compared to the single arms (Fig. Suppl. 3B). In this regard, B16-F10 has been largely described in the literature as a standard syngeneic murine model showing a fast tumor growing and aggressiveness. However, this cell line is poorly immunogenic and lacks any mutational burden occurring in humans^18^.

### NextA modulates the expression of immune-regulatory mediators in tumor cells

As mentioned earlier, anti-PD-1 treatment is associated with an up-regulation of PD-L1 in patients^15^ and animal studies (Fig. 1C). Our initial working hypothesis stated that targeting HDAC6 enzymatic activity could improve the therapeutic effect of anti-PD-1 by decreasing the up-regulation of immune-suppressive mediators such as PD-L1, PD-L2, B7-H3, and B7-H4 in tumor cells. Moreover, based on our previous findings, we also expected HDAC6i induce changes in the TME, namely improved antigen presentation, decreased anti-inflammatory cytokines, and changes in the composition of TILs. First, we evaluated SM1 melanoma tumor cells collected from mice treated with vehicle control, anti-PD-1 antibody, NextA, or a combination of both agents for expression of PD-L1 and other immune-regulatory surface markers by flow cytometry. As expected, the presence of PD-L1 (Fig. 2D) was slightly up-regulated in the anti-PD-1 arm, and its expression was significantly reduced in the NextA and combination treatment arms. This result was corroborated by immunoblot studies of total PD-L1 protein (Fig. 2I), and qRT-PCR analysis of PD-L1 mRNA levels (Fig. Suppl. 4A). Likewise, we observed a slight increase in the expression of PD-L2 in the anti-PD-1 condition by both flow cytometry (Fig. 2E) and mRNA analysis (Fig. Suppl. 4B). While the PD-L2 expression was not significantly reduced in the NextA and combined conditions, we observed that the combination arm prevented its up-regulation induced by anti-PD-1 blockade. We previously observed that the total levels of B7-H3 and B7-H4 were down-regulated in HDAC6KO tissue (Fig. Suppl. 1C). However, these surface markers were not affected in any of the aforementioned experimental arms (Fig. Suppl. 3M,N). Additionally, we evaluated whether the presence of the co-stimulatory protein OX40-L was affected under our experimental conditions. Interestingly, OX40-L expression was found to be diminished in the anti-PD-1 group but was restored in the NextA and combination groups as evidenced by flow cytometry (Fig. 2F) and mRNA analyses (Fig. Suppl. 4D).

Treatment with HDAC6i has been shown to induce the expression of MHC class I in tumor cells^11^. A similar outcome was observed in our NextA-treated experimental group, with a further increase in MHC class I expression being noted in the combination arm (Fig. 2G). Similarly, we observed an increase in the presence of MHC class II in the combination group, while no significant changes occurred in any of the independent arms (Fig. 2H). Other immunomodulatory markers such as galectin-9, ICOS-L, CD80, CD86, CD70, and HVEM did not show any significant changes in any experimental condition (Suppl. 4G–L**)**.

### HDAC6 intervention reduces the anti-inflammatory phenotype of TAMs

In general, macrophages can be polarized to M1 or M2 macrophages. M1-polarized macrophages produce pro-inflammatory and immune-stimulatory mediators. In contrast, M2 macrophages are often associated with a poor overall prognosis as they play a critical role in negatively modulating the immune response, ultimately supporting tumor growth. M2 macrophages have been shown to promote the initiation, proliferation, invasion, and metastasis of solid tumors, and facilitate the progression of tumor growth by stimulating angiogenesis through secretion of the enzymes plasmin, uPA, matrix metalloproteinases (MMPs) and cathepsin B^19^. Furthermore, M2 macrophages inhibit the anti-tumor responses of cytotoxic T cells and induce the development/recruitment of immunosuppressive regulatory T cells to the TME. Tumor-associated macrophages (TAMs) found in the tumor microenvironment have previously been described as predominantly having an M2 phenotype^20^, but it has been shown that they can express both M1 and M2 polarization hallmarks^21^. Importantly, reprograming TAMs to an M1 phenotype in order to promote anti-tumor activity is a current therapeutic strategy to improve anti-tumor immune responses^22^. Therefore, we wanted to know whether the phenotype of macrophages found in tumor samples was affected after *in vivo* treatment with NextA. First, we measured the percentage of M1 (F4/80+, H2+, CD80+) and M2 (F4/80+, CD206+) TAMs, respectively. Although we observed minor differences in the M1 phenotype across all conditions, the M2 population was notably enhanced in the anti-PD-1 treatment. On the other hand, the addition of NextA not only diminished the M2 phenotype but also counteracted the effect of anti-PD-1 in the combination arm (Fig. 2J). Of note, while the absolute number of macrophages was increased in the anti-PD-1 condition (data not shown), the ratio M1/M2 in the NextA treatment group was the highest across all conditions. To further investigate the effect of NextA and anti-PD-1 on the inflammatory cytokine milieu, we evaluated the intratumoral cytokine composition under each experimental condition. We observed a considerable augmentation in the levels of pro-inflammatory cytokines IFNγ and IL-2 across all treatment conditions when compared to the non-treated group (Fig. 2K,L). Nonetheless, the levels of IL-12 were only up-regulated in the anti-PD-1 condition (Fig. 2M). Additionally, the presence of the anti-inflammatory cytokines IL-10 and TGFβ was increased in the anti-PD-1 condition and slightly diminished in the NextA and combination arms (Fig. 2N,O), suggesting that NextA neutralized the anti-PD-1 effect over the production of these particular cytokines.

### Pre-treatment with NextA further improves anti-tumor immune response when using anti-PD-1 immune checkpoint blockade

**Figure 3:**
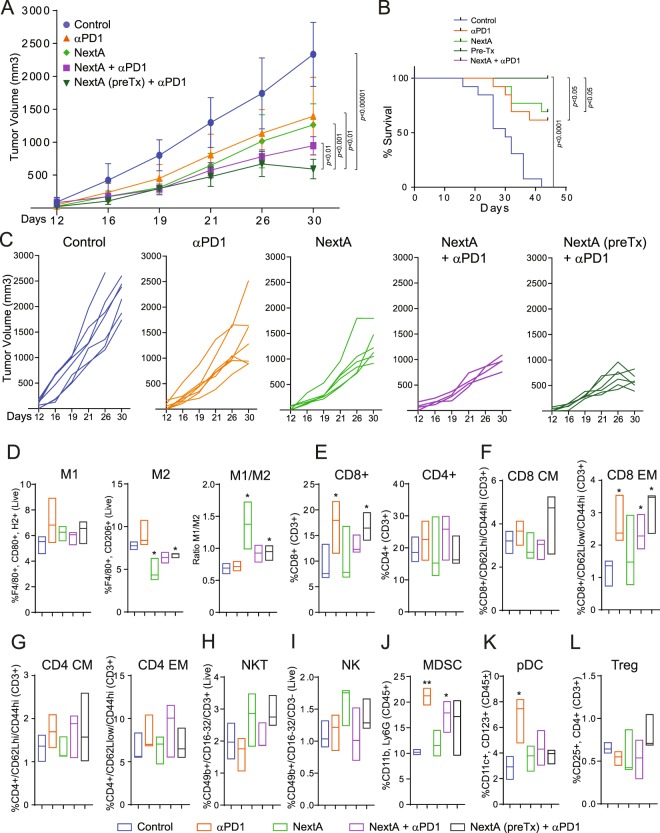
Pre-treatment with NextA further improves anti-tumor immune response when using anti-PD-1 immune checkpoint blockade. (**A**) C57BL/6 mice were subcutaneously injected with 1 × 10^6^ SM1 murine melanoma tumor cells. Mice were treated with a vehicle control, 15 mg/kg anti-PD-1, 25 mg/kg NextA, or a combination of both agents for 30 days. The NextA pre-treatment noted in the figure started 10 days before other conditions. (**B**) Kaplan-Meier survival plot of the previous study. (**C**) Individual group plots representation for the previous study. (**D**) Tumors were collected at the end point and the presence of M1 and M2 surface markers were evaluated by flow cytometry. M1/M2 ratios were calculated from three independent populations from M1 and M2. Similarly, tumors were screened by flow cytometry to evaluate the infiltration of CD8+, CD4+, NKT, NK, MDSC, pDC and Tregs (**E**–**L**), and specific markers of memory formation were evaluated as noted in the figure (**F,G**). Representative panels for all conditions are shown in Supplementary Figs 8 and 9.

Our results indicate that the single-agent treatment of SM1-challenged mice with NextA not only reduces the expression of several immunosuppressive mediators but also significantly decreases the presence of M2 macrophages in the TME. This outcome was also observed when combining this drug with anti-PD-1 antibody, suggesting that the effect of NextA could operate independently of the immune checkpoint blockade, and potentially prepare the tumor microenvironment in ways that further enhance the effect of anti-PD-1 blockade treatment. To test this hypothesis, we evaluated the anti-tumor activity in mice pre-treated with NextA for 10 days prior to combinatorial treatment of anti-PD-1 antibody and NextA. In order to evaluate the “immune priming” role of NextA, we needed to adjust the drug administration schedule of the single and combination treatments. Thus, we began the single NextA and the pre-treatment groups 4 days after tumor inoculation and waited for 10 days before starting the rest of the study arms. In a 30-day lapse study, single therapy with either anti-PD-1 antibody or NextA, or, otherwise, the standard combination treatment decreased tumor growth as previously determined (Fig. 3A–C). As hypothesized, pre-treatment with NextA further reduced tumor growth when compared to the combination regimen. Additionally, the pre-treatment arm maintained a 100% survival when compared to the simultaneous combination treatment (Fig. 3B).

Next, we evaluated whether the pre-treatment with NextA induced any changes in the intratumoral macrophage composition. As observed previously, treatment with Nexturastat alone strongly reduced the M2 phenotype and increased the M1/M2 ratio in these tumors (Fig. 3D). Although we did not observe a significant reduction of M2 macrophages in the pre-treatment condition, the final M1/M2 ratio was significantly superior to the control group.

### The combination of NextA and anti-PD-1 enhances T cell infiltration in tumors

High infiltration of CD8+ T cells is considered a marker of clinical response during anti-PD-1 treatment in several malignancies such as melanoma^23^, breast cancer^24^, glioblastoma^25^, synovial sarcoma^26^, and several other malignancies. Similarly, NK infiltration has been associated with non-progressive disease in NSCLC^27^. A number of other potential biomarkers have been analyzed in melanoma, including the presence of specific populations of immune cells infiltrated in the tumor, lymph nodes, and peripheral blood^23^. Taking the above into consideration, we evaluated the compositions of infiltrated immune cells in end-point (30 days) tumor nodules. The flow cytometry strategies and panel design to evaluate each cellular population are shown in Supplementary Figs 7–10. We first analyzed tumors for the presence of infiltrated CD3+ T cells. While CD8+ T cells were notably increased in mice treated with anti-PD-1 blockade, the CD4+ population was found to be unaffected (Fig. 3E). Similar outcomes have been reported from clinical trials, where the infiltration of CD8+ T cells in tumors often correlates with better patient outcome and response to anti-PD-1 treatment^4^. Conversely, no major changes in the CD4+ population have been observed between responder and non-responder patients after the same therapeutic regimen^4^. Of note, while treatment with NextA alone had a minimal effect on the infiltration of CD8+ cells, the combination with anti-PD-1 enhanced the footprint of CD8+ lymphocytes in tumors. Although the percentage of total CD8+ cells appeared to be unaffected by the combination of NextA and anti-PD-1 antibody, breaking down the populations into central memory vs. effector memory showed that the combination of both agents, pre-treatment with NextA as well as single therapy with anti-PD1 antibody enhances the effector memory T cell compartment within the tumor tissue (Fig. 3F,G).

In a further characterization, we also observed a slight increase in the infiltration of NK and NKT cells in the single and pre-treatment combination arms including NextA (Fig. 3H,I), suggesting that HDAC6 could also be involved in the intra-tumoral recruitment of these cell types. On the other hand, MDSC and pDC populations also showed a significant tumor infiltration within the anti-PD-1 treatment group, but no evident changes were observed in the NextA-treated experimental group (Fig. 3J,K). Interestingly, the NextA combination arms counteracted the effect of anti-PD-1 treatment, diminishing the presence of pDC to basal levels, and slightly reducing the MDSC population. Given the tumor-promoting roles of pDC in melanoma described earlier^28^, this decrease may be a promising outcome for HDAC6 inhibition. An additional screening of Tregs demonstrated no significant changes under all tested conditions (Fig. 3L).

### The anti-tumor effect of HDAC6i requires an intact host immune system

**Figure 4:**
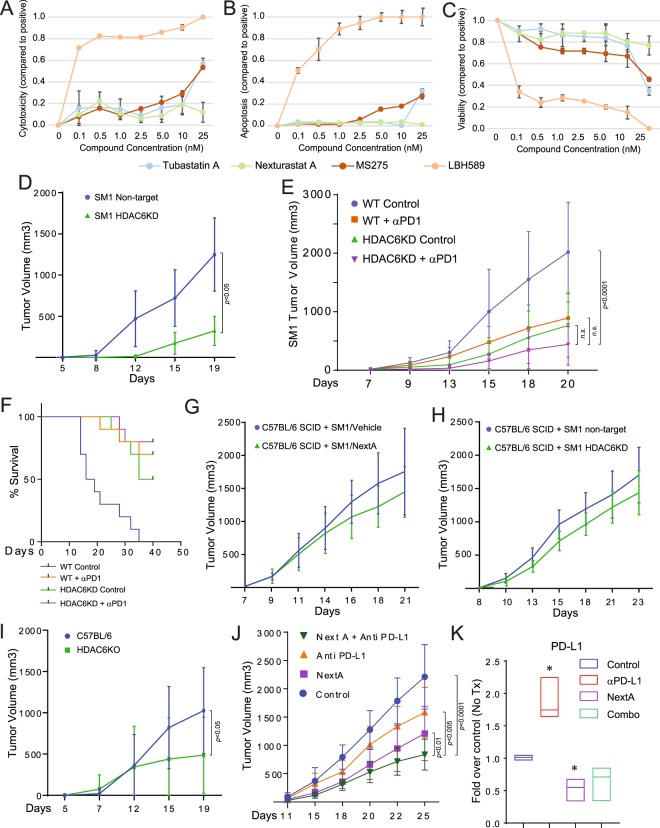
The anti-tumor effect of HDAC6i requires an intact host immune system. SM1 cells were treated with different concentrations of Tubastatin A, LBH589, MS275 and NextA for 24hrs and then evaluated for cytotoxicity (**A**), apoptosis (**B**) and viability (**C**) using Apotox-Glo® Triplex Assay. (**D**) C57BL/6 mice were subcutaneously injected with 1 × 10^6^ SM1 murine melanoma cells lacking HDAC6 (HDAC6KD) or control cells (non-target). (**E**) C57BL/6 mice were subcutaneously injected with 1 × 10^6^ HDAC6KD or non-target SM1 murine melanoma cells. Mice were then treated with anti-PD-1 (15 mg/kg) 5 times a week. (**F**) Kaplan-Meier survival plot of the previous study. (**G**) C57BL/6 SCID mice were subcutaneously injected with 1 × 10^6^ SM1 murine melanoma cells and then treated with 25 mg/kg NextA. Tumor growth was monitored for 21 days. (**H**) C57BL/6 SCID mice were subcutaneously injected with 1 × 10^6^ HDAC6KD SM1 or non-target murine melanoma cells. Tumor growth was monitored for 23 days. (**I**) HDAC6−/− mice were subcutaneously injected with 1 × 10^6^ SM1 murine melanoma tumor cells. Tumor growth was monitored for 19 days. (**J**) C57BL/6 mice were subcutaneously injected with 1 × 10^6^ SM1 murine melanoma tumor cells. Mice were treated with a vehicle control, 15 mg/kg anti-PD-L1, 25 mg/kg NextA, or a combination of both agents for 25 days. (**K**) The expression of PD-L1 from tumor samples was evaluated qRT-PCR.

The anti-proliferative and pro-apoptotic effect of non-specific HDACi has been extensively reported^7^. However, selective HDAC6i have shown to minimally affect the viability of tumor cells^29^. In this regard, treatment with NextA (25 μM) demonstrated minimal cytotoxicity (Fig. 4A), and negligible changes in viability (Fig. 4B) and apoptosis (Fig. 4C) when compared to the selective HDAC6i Tubastatin A, the pan HDACi LBH589, and the class I inhibitor MS275.

Since HDAC6i did not show anti-tumor effects in melanoma cells cultured *in vitro*, we wanted to evaluate if the same situation occurred in tumor cells in an *in vivo* context. To answer this question, we challenged wild-type C57BL/6 mice (fully functional HDAC6 activity) with SM1 melanoma cells lacking HDAC6 (HDAC6KD). As shown in Fig. 4D, the absence of HDAC6 in tumor cells significantly reduced tumor growth. Although the analysis of the endpoint tumors indicated a slight increase in the infiltration of CD8+ T cells in the HDAC6KD group, this increment was not significant (Fig. Suppl. 5B). Additionally, we observed a slight reduction in the cell surface expression of the immunosuppressive receptors LAG3 and TIM3 in both CD4+ and CD8+ populations, with only the reduction of TIM3 in CD4+ cells found to be significant. However, PD-1 expression was only reduced in CD4+ cells (Fig. Suppl. 5C). Then, we evaluated whether HDAC6KD SM1 mouse melanoma cells were more sensitive *in vivo* to the anti-tumor effect induced by anti-PD-1 immune checkpoint blockade. As expected, each independent condition, HDAC6KD, and anti-PD-1 antibody were able to reduce tumor growth (Fig. 4E and Suppl. 5A) and improve survival (Fig. 4F) when compared to the non-treated group. However, no statistically significant difference on tumor growth was found between the combination of tumor-targeted HDAC6KD and anti-PD-1 relative to each individual arm, indicating that the abrogation of HDAC6 in SM1-derived tumors was not enough to further improve the anti-tumor effect of the anti-PD-1 blockade.

To evaluate the participation of the host immune system in the anti-tumor effect induced by HDAC6i, we treated immune deficient SCID (NOD.CB17-Prkdc^scid^/NcrCrl) mice with NextA or vehicle. As shown in Fig. 4G, the treatment with NextA minimally affected tumor growth. Similarly, we did not observe major effects in tumor growth when inoculated SM1 HDAC6KD into SCID mice (Fig. 4H). These results demonstrated that the anti-tumor effect of HDAC6i was also mediated by immune host components, in addition to the already determined effect over immune modulators in tumor cells. To further explore this possibility, we challenged HDAC6−/− mice with wild type SM1 cells which exhibited slower growth than in wild type mice (Fig. 4I). The analysis of the end-point tumors evidenced no changes in the infiltration of CD8+ and CD4+ T cells (Fig. Suppl. 5D) and no significant changes were observed in the expression of PD-1, LAG3 and TIM3 (Fig. Suppl. 5E). Thus, the collective analysis of the previous *in vivo* experiments suggests that although the anti-tumor effect of HDAC6i is mainly mediated by a direct effect over tumor cells, there are also some specific host components needed to further increase this anti-tumor activity. These results suggested that the downregulation of PD-L1 expression by HDAC6i was not the only mechanism involved in the improved anti-tumor effect when combining anti-PD-1 and NextA. To evaluate this possibility, we performed an *in vivo* combination study using NextA plus anti-PD-L1 blocking antibody. Briefly, if the main effect of HDAC6i was directly mediated by the abrogation of PD-L1, we should expect no further improvement in anti-tumor activity when blocking PD-L1 in tumor cells. However, we observed a significant reduction in tumor growth when comparing this combination against each independent experimental arm (Fig. 4J). The subsequent analysis of end-point tumors showed that the treatment with anti-PD-L1 antibody induced the expression of PD-L1 almost in the same magnitude previously observed with the treatment with anti-PD-1 antibody. Moreover, the treatment with NextA was able to reverse the up-regulation of PD-L1 expression (Fig. 4K).

### HDAC6i enhance the pro-inflammatory phenotype of macrophages

**Figure 5:**
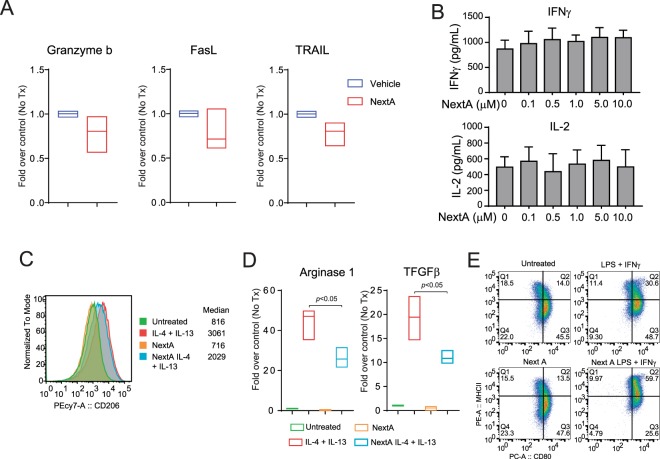
Combination treatment significantly decreases the M2 macrophage phenotype and immunosuppressive cytokines in the tumor microenvironment. (**A**) NK cells isolated from spleen were incubated with NextA and then evaluated for the expression of Granzyme b, FasL, and TRAIL by qRT-PCR (**B**) Murine splenocytes were incubated for 1 hour in the presence or absence of NextA before activation with anti-CD3/28 beads. The presence of IFNγ and IL-2 was measured by ELISA after 24hrs. Murine peritoneal elicited macrophages (PEM) primary cells were treated with IL-4(20 ng/mL) and IL-13(20 ng/mL) in the presence or absence of NextA (5 μM). After 24hrs, the expression of CD206 (**C**), arginase 1 and TGFβ (**D**) were measured by flow cytometry and qRT-PCR, respectively. PEM cells were treated with LPS (100 ng/mL) and IFNγ (10 ng/mL) in the presence or absence of NextA (5 μM). After 24hrs, the expression of MHC class II and CD80 were measured by flow cytometry (**E**).

We observed a slight increase in the tumor infiltration of NK cells upon treatment with NextA across all *in vivo* experiments. Therefore, we wanted to investigate whether the inhibition of HDAC6 was able to modify the phenotype of these immune cells. We harvested fresh NK cells from non-tumor bearing wild type C57BL/6 mice and treated them with NextA *in vitro* to investigate the reported markers for NK cell activity and cytotoxicity, such as Granzyme B, TRAIL, and Fas ligand. Granzyme B has been described as important soluble mediator for cytotoxicity whereas FasL and TRAIL are related to apoptotic cell death and NK cell activation^30^. Although we observed an enhanced tumor infiltration of NK cells in all *in vivo* conditions using NextA, the aforementioned activation markers were minimally affected in isolated NK cells treated with this drug (Fig. 5A), suggesting that the enhanced NK infiltration observed after NextA treatment could be mediated by other indirect mechanisms.

Similarly, although we did not observe an important increase in the infiltration of CD8+ and CD4+ T cells upon *in vivo* treatment with NextA, we wanted to evaluate if HDAC6i had any effect on isolated T cells. Using CD3+/CD28+ T cells isolated from immune competent mice, we observed that NextA did not induce major changes in the production of either IFNγ and IL2 (Fig. 5B), implying that the anti-tumor activity of HDAC6i was not mediated by the direct action of this drug on T cells.

Another important immune effect induced by HDAC6i *in vivo* treatment was the augmentation of the ratio M1/M2 phenotype of intratumoral macrophages. To further investigate the effect of NextA on macrophages, we isolated murine peritoneal elicited macrophages (PEM) primary cells and polarized them to M1 and M2 in the presence or absence of NextA. As described previously^31^, the M2 marker CD206 was increased after treatment with IL-4(20 ng/mL) and IL-13(20 ng/mL). However, this up-regulation was diminished in the presence of NextA(5 μM) (Fig. 5C). Additionally, the production of the M2 activated genes arginase 1 (Arg1) and TGFβ was significantly decreased in the presence of NextA (Fig. 5D). We also wanted to evaluate the effect of NextA over the M1 phenotype of primary macrophages by treating freshly isolated PEM with LPS (100 ng/mL) and IFNγ (10 ng/mL) as described previously^31^. Although the M1 phenotype was greatly increased under this double stimulus (14 to 30.6 double positive), this effect was further increased to 59.7 double positive in the presence of NextA (Fig. 5E), suggesting that the inhibition of HDAC6 enzymatic activity improves the pro-inflammatory phenotype of murine PEMs in addition to its negative effect over the M2 polarization. These results align with previous reports from our lab indicating that PEM treated with HDAC6i trigger a better activation of HA-specific CD4+ T-cells as evidenced by increased production of IFNγ^32^.

The important role of the macrophage composition in anti-tumor immune responses has been extensively documented in several types of cancer^33,34^. In order to evaluate if the macrophage phenotype could have any influence in the tumor growth in our specific syngeneic tumor model, we challenged C57BL/6 mice with SM1 melanoma and treated mice with clodrosomes, or vehicle encapsosomes, intratumorally after the tumor reached 400 mm^3^ for two weeks. As reported previously^33,34^, the depletion of macrophages was accompanied by a decrease in the presence of DC and no effect over the composition of T cells (Suppl. Fig. 6A). As hypothesized, the tumor growth of the mice treated with clodrosomes was significantly diminished (Suppl. Fig. 6B), suggesting the pro-tumoral component of macrophages in the TME was a predominant factor favoring tumor survival.

## Discussion

A number of pre-clinical and clinical studies have reported that the expression of PD-L1 increases upon anti-PD-1 immune check-point blockade therapies^35^. Accordingly, preliminary studies in tumor biopsies of anti-PD-1 treated melanoma patients suggest that the up-regulation of PD-L1 early during the therapy could be used as a premature tool to predict response^36^. Although the expression of PD-L1 could be used as a biomarker of response in immunotherapy, the expression of this protein before the start of treatment seems to be variable and no clear evidence has been found to assign its expression as a universal predictive biomarker. Nevertheless, its evaluation has been recently implemented as a potential biomarker for specific types of malignancies^37^. Regardless of the potential use of PD-L1 as an immunotherapeutic biomarker in patients undergoing immunotherapy, the up-regulation of this protein before immunotherapy treatments has been associated with poor prognosis in several types of malignancies^38^. Additionally, it is imperative to mention the potential significance of other immune-regulatory biomarkers while analyzing the expression of PD-L1. This highlights the importance of the composition of tumor-infiltrating lymphocytes (TILs). In particular, since the infiltration of CD8+ T cells in addition to the upregulation of PD-L1 has been reported as a promising biomarker of response to PD-1 immune checkpoint blockade^4,26^.

Our initial premise stated that the up-regulation of PD-L1 upon immune check-point blockade treatment is a direct consequence of the high levels of pro-inflammatory cytokines such as IFNγ. To prove this hypothesis, we evaluated the expression of PD-L1 in the presence of IFNγ blocking antibody, which resulted in a complete abrogation of PD-L1 expression. Interestingly, we observed similar experimental outcome in the presence of the HDAC6i NextA, suggesting that the intervention of this particular HDAC could be an effective way to neutralize the upregulation of the immunosuppressive pathways triggered by IFNγ.

In theory, the up-regulation of PD-L1 due to high levels of IFNγ should not have a critical impact upon anti-PD-1 therapies, as this specific pathway is already neutralized by the anti-PD-1 blocking antibody. However, other immunosuppressive check-point molecules such as galectin-9^39^ and PD-L2^40^ have also been reported to be affected by IFNγ. Thus, the activation of these immunosuppressive pathways could counteract the efficacy of the treatment with anti-PD-1 blocking antibody. In this context, the use of HDAC6i could neutralize this immunosuppressive negative feedback and concurrently improve anti-tumor immune responses by modifying the phenotype and function of other cellular components present in the TME^32,41^. In addition to these reported activities, we have found that selective HDAC6i have minimal cytotoxicity when tested *in vitro* and *in vivo* models; characteristics often encountered in pan-HDACi and other non-selective HDACi. However, a number of partially selective HDAC6i have been reported to have an impact on cytotoxicity and/or cell cycle arrest over tumor cells^41^. Nevertheless, there is a possibility that these inhibitors could be less specific and also target other HDACs, and these off-target effects contribute to the above-mentioned effects on cellular proliferation. This is particularly relevant for some HDACi targeting simultaneously HDAC6 and class I HDACs such as ACY241^42^ and ACY1215^43^.

HDAC6 has been also reported to play a role in signaling pathways relevant in melanoma, such as those involving p53 (73), PTEN (35), and different proteins in the MAPK pathway, such as Ras^44^ and Erk1/2^45^. Specifically, HDAC6 deacetylates p53 at Lys381/382, and levels of HDAC6 in the nucleus inversely correlate with the expression of p53 target genes (73). In addition, HDAC6 plays a role in p53 degradation by inhibiting its interaction with p300 and promoting its interaction with Mdm2^46^. Therefore, treatment with selective HDAC6 inhibitors could decrease p53 degradation in tumors bearing wild-type p53. Furthermore, HDAC6 inhibition enhances the translocation of PTEN to the membrane, thus reducing AKT phosphorylation and cell growth^47^. Tumor cells bear mutations that inactivate p53 or PTEN and activate different components of the MAPK pathways, thus suggesting that HDAC6 regulates the activity of both tumor suppressor genes and oncogenes. Although HDAC6i seems to be involved in the regulation of important oncogenic pathways, it seems like its main anti-tumoral activity is through the regulation of immune-related pathways. This becomes evident when evaluating the effect of HDAC6i in immunocompromised mice, which do not show any significant improvement in the anti-tumor activity when treated with these inhibitors, demonstrating that the anti-tumor activity of HDAC6i requires an intact adaptive immune system. Although the selective silencing of HDAC6 in tumors reduces tumor growth in immunocompetent mice, this effect was not statistically significant. Moreover, the fact that the SM1 tumor growth in HDAC6KO mice is reduced, suggests that the intervention of HDAC6 in the host is also an important component in the anti-tumor activity observed in the systemic use of HDAC6i. Additionally, an intriguing pre-clinical observation made in immune-competent mice challenged with melanoma demonstrates that acquired resistance to immune checkpoint blockade is reversed by inhibiting JAK1/JAK2 signaling^48^.

Our results from the combinatorial studies of NextA and anti-PD-1 blocking antibody clearly demonstrate an improvement in the reduction of tumor size and survival, which was associated with a reduction in the expression of several immunosuppressive mediators in tumors, both of which fully support our initial hypothesis. Additionally, in all the performed replicates (8 independent experiments) we observed between 15–40% of the mice undergoing total remission in the combination arm before the end of the study, while in the single arm treatment with anti-PD-1 we observed total remission in 0–10% of the replicates we performed which occurred before day 36 marking the treatment endpoint (data not shown).

Differentiation and maturation of myeloid cells, in response to micro-environmental signaling, are regulated by transcription factors governed the “epigenetic landscape” that determines the phenotype and function of these cells. In further screenings of end-point tumors, we observed a significant decrease in the presence of M2 macrophages (inhibitory sub-population) in the combination therapy, which we believe is a direct consequence of HDAC6i in macrophages. This was demonstrated by the potent effect of NextA over the polarization of macrophages in isolated *ex vivo* assays performed in murine PEM and the macrophage cell line RAW264.7. Importantly, it has been proposed that several of the physiological effects observed as *irAEs*, including cytokine storm, are mediated by tumor-associated macrophages (TAM)^49^, which are predominantly M2 macrophages and frequently up-regulated after anti-PD-1 treatment^50^. Our results show a significantly lower population of the suppressive M2 phenotype of macrophages. Such a dramatic decrease in this population of immune cells likely plays a role in the antitumor effects observed in our mouse cohorts. The immunomodulatory role of HDAC6 in regulating IL-10 production by macrophages has been reported previously^32,51^. These early reports align with the findings reported here; demonstrating a pivotal role of HDAC6 in controlling the inflammatory environment in the TME, tilting the balance to a pro-inflammatory landscape and favor anti-tumor immune responses. Thus, our results suggest that the pre-treatment with HDAC6i could also contribute to control *irAEs* triggered by the immune check-point blockade.

Although we observed an improvement of the infiltration of CD8+ and NK cells in the combination arm, these changes could be an indirect consequence of the changes in the intra-tumoral cytokine milieu induced by the aforementioned effects over TAMs and tumor cells, and further investigation is needed to provide a conclusive mechanism of action. Given the described interaction of NK cells being capable of eliminating intratumoral M2 macrophages^52^, there could also be an indirect effect induced by the addition of HDAC6i. In recent years, NK cells have been identified to play a critical role in immunosurveillance and host defense mechanism against malignancies^53^. Enhanced NK infiltration has even been associated with a better prognosis for survival in breast cancer^54^ and metastatic prostate cancer^55^. Interestingly, the treatment of NK cells with the HDACi valproic acid (a wide spectrum HDACi), was shown to increase the expression of the NK activation marker NKG2D receptor through the suppression of STAT3, a well-known target for HDAC6^56^. High infiltration of CD8+ and activated NK has been previously associated with better clinical outcomes and overall survival in lung cancer and melanoma patients under anti-PD-1 treatment^27^.

**Figure 6:**
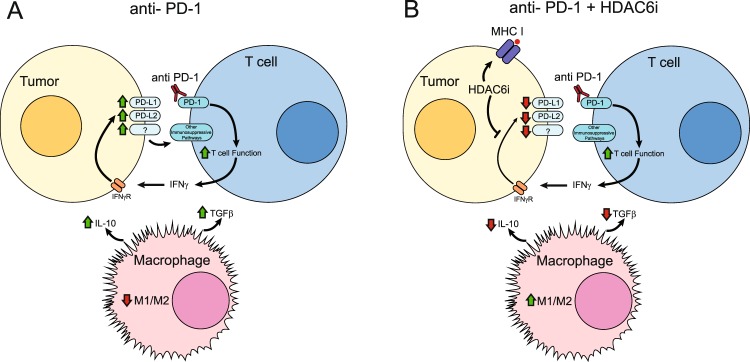
Schematic representation of the role of HDAC6 in the TME. Combination treatment of anti-PD-1 blocking antibody and selective HDAC6i. (**A**) Treatment with anti-PD-1 antibody enhances T cell function and the release of pro-inflammatory cytokines. These mediators induce the production of several immunosuppressive proteins in tumor cells, including PD-L1 and PD-L2. Additionally, anti-PD-1 treatment reduces the pro-inflammatory phenotype of macrophages. (**B**) The addition of selective HDAC6i neutralizes the negative feedback induced by IFNγ and de-activates immune inhibitory check-point signals.

Finally, a schematic representation of our findings is presented in Fig. 6. In conclusion, although the treatment with anti-PD-1 antibody reduces tumor growth in mice challenged with melanoma cells, we have found that this treatment induces the expression of PD-L1 and other immunosuppressive mediators via up-regulation of IFNγ. Additionally, the anti-PD-1 treatment triggers profound changes in the macrophage phenotype, diminishing their inflammatory phenotype (Fig. 6A). On the other side, the incorporation of selective HDAC6i to the anti-PD-1 therapy was able to reduce the expression of immunosuppressive proteins and increase the inflammatory phenotype of macrophages in the TME (Fig. 6B). Our studies using a pre-treatment with NextA demonstrate a significant improvement in the anti-tumor effects of anti-PD-1 immune checkpoint blockade therapy.

## Materials and Methods

### Mice

Experiments involving mice were performed in accordance with approved protocols by the Institutional Care and Use Committee (IACUC) at The George Washington University (Protocol A354). C57BL/6 mice were obtained from the Charles River Laboratories (Wilmington, Massachusetts, USA), NOD.CB17-Prkdc^scid^/NcrCrl were obtained from Jackson Laboratory (Bar Harbor, ME, USA), and the HDAC6KO (H-2b) were kindly provided by Dr. P. Matthias (Friedrich Miescher Institute for Biomedical Research, Basel, Switzerland)^13^. For *in vivo* tumor studies, mice were subcutaneously injected into a shaved flank with 1.0 × 10^6^ melanoma cells suspended in 100 μL 1X phosphate buffered saline (PBS) (Corning, 21–040-CV). Treatment began as soon as the tumors were palpable or as indicated in particular experiments and ended when tumor reached 2500 mm^3^ (point considered as dead in survival analysis). Mice were injected with NextA, anti-mouse PD-1 (BioXCell, BE0033-2), anti-mouse PD-L1 (BioXCell, BE0101), or combinations as indicated for each experiment. Control mice were injected with 100 μL 1X PBS as a vehicle control. Mice were injected five days a week until tumors in the control group reached maximum size according to our IACUC protocol. Tumor volume was calculated from caliper measurements by the formula L × W^2^/2. All the *in vivo* studies were performed with tumor cells passaged *in vivo* (mouse to mouse) at least five times before the tumor challenge experiment.

All animal studies were done considering the toxicity for each individual agent and using the dose range suggested by the manufacturers. However, we performed routine monitoring to evaluate early signs of toxicity in all conditions. Particular focus was given to mortality, body weight, food consumption, and postmortem evaluation including gross visual examination of organs at all tested conditions. All animal studies were done in accordance with all guidelines and regulations allowed by the IACUC protocols approved by the animal facility at the George Washington University.

### Cell culture

#### *In vivo* tumors

The SM1 cell line was obtained from the laboratory of Dr. A. Ribas at the University of California Los Angeles^17^. SM1 cells were passaged *in vivo*; directly from mouse to mouse for at least five passages. Tumor clones were grown and selected for optimal and consistent growth rate. When preparing cells for tumor injection, mice with tumors measuring approximately 7 mm × 7 mm were euthanized. Tumors were extracted and processed under sterile conditions. Cell concentration was determined and adjusted to the appropriate injectable amount per mouse (1.0 × 10^6^ per 100 μL). These cells were immediately injected into animal study subjects, as described above. Any excess cells from the tumor processing were immediately frozen in 90% Fetal Bovine Serum (FBS) (Serum Source, FB02–500HI) with 10% Dimethyl sulfoxide (DMSO) (Sigma-Aldrich, D2650) and stored in liquid nitrogen for future use.

#### Tumor/T cell co-culture

SM1 melanoma cells were cultured in a six-well plate to form a monolayer to 70% confluency. Splenocytes from C57BL/6 mice were harvested and homogenized. CD3+ cells were isolated by EasySep magnetic kit (StemCell, 19851 A) through negative selection. Cells were checked for viability to > 85%. CD3+ splenocytes then were stimulated with CD3/28 magnetic dynabeads (Thermo Fisher Scientific, 11456D) overnight in complete RPMI 1640 media (Gibco, 11875101). The stimulated T cells were then layered onto the SM1-monolayer for 24 hours. Anti-PD1 antibody (BioXCell, BE0033-2) was then added to the culture at a final concentration of 1ug/ml and cultured for another 24 hours. Anti-mouse IFNγ antibody (PBL Assay Science, 22500-1, rabbit serum;) was then added to the culture at two different dilutions of 1:100 and 1:1000 relative to the total volume of culture media. In a separate well, NextA was added to a final concentration of 2.5 and 5 μM. Cells and supernatant were collected after 24 hours of incubation for detection of PD-L1 expression (Qiagen, 74104) and secreted levels of IFNγ by ELISA (R&D Systems, MIF00).

#### Mouse peritoneal elicited macrophage (PEM) isolation and polarization

C57BL/6 mice were injected intraperitoneally with 3% thioglycolate and humanely euthanized four days post-injection. The peritoneal macrophages were collected under sterile conditions and cultured in RPMI 1640 with 10% Fetal Bovine Serum (FBS), 1% MEM non-essential amino acids (NEAA) (Corning, 25-025-Cl) and 1% Penicillin-Streptomycin (Corning, 30-002-CI) and grown to 70–80% confluency. To polarize into M1-like macrophages, 100 ng/mL lipopolysaccharide (LPS) (Sigma-Aldrich, L2880) and 20 ng/mL IFNγ (Biolegend, 575304) were added to the PEM culture and incubated with 5 μM NextA for a total for 24 hours. To polarize into M2-like macrophages, 20 ng/mL IL-4 (Peprotech, 214-14) and 20 ng/mL IL-13 (Biolegend, 575902) were added to the PEM culture and incubated with 5 uM NextA.

#### Cellular viability and Apoptosis assays

Viability and apoptosis were measured using *ApoTox-Glo Triplex Assay*® (Promega, G6321). Murine melanoma cells were treated with individual HDACi along with the protocol recommended assay controls. Following the manufacturer’s protocol, Viability/Cytotoxicity reagents were added, and fluorescence was measured at specific wavelengths – i.e., 400Ex/505Em (viability) and 485Ex/520Em (cytotoxicity). Next, the Caspase 3/7 reagent was added, and after incubation, luminescence was measured at Lm578 (apoptosis). Assay measurements were collected using the SpectraMax multi-mode microplate reader. During our analyses, LBH was used as the control compound.

#### T cell cytokine analysis

Mouse spleens and lymph nodes (LN) were harvested and prepped into pertinent single cell suspensions. Mouse CD3 T cells were then negatively selected from the entire splenocyte and LN cell populations respectively, using the EasySep magnetic kit. Isolated CD3 T cells were incubated for 1 hour in the presence or absence of HDACi prior to activation with anti-CD3/28 dynabeads (Thermofisher) in 96-well plates. Cell supernatants were collected at 48 hours and 72 hours post T cell activation, briefly centrifuged for cellular and dynabeads removal and subsequently analyzed by ELISA.

NK cells were isolated from mouse spleen using the MagCellect mouse NK cell isolation kit (R&D Systems, Cat# MAGM210). Briefly, freshly harvested spleens from C57BL/6 male mice (aged 8 weeks) were gently homogenized and treated with ACK lysis buffer (Thermo Fisher Scientific; A1049201). Washed and aliquoted cells were then treated sequentially with biotinylated antibody cocktail and streptavidin ferrofluid followed by magnetic separation. The eluted cells after negative selection were checked for viability (~78%).

A smaller aliquot of the cells were compared with unfractionated splenocytes to confirm NK cell-enrichment. For this, the cell aliquots were stained for viability; surface bound CD49b and CD16/32 expression (full description in the flow cytometry section).

Cells were then aliquoted in six well plate and cultured overnight with 20 ng/ml IL2 (Biolegend, Cat# 575404) and 10 ng/ml IL15 (Biolegend, Cat# 566302). Cells were treated with 0.5μg/ml NextA for six hours, followed by RNA extraction.

### Macrophage depletion

One million SM1 cells were injected subcutaneously in the flanks of C57/Bl6 mice. When tumors became palpable, macrophage depletion was performed intratumoral injection of 200 μl of Clodrosomes (clodronate-encapsulated liposomes) or Encapsomes (PBS liposomes) (Encapsula NanoSciences LLC, Brentwood, TN) for two weeks.

### HDACi and anti-PD-1

The HDAC6 selective inhibitor Nexturastat A (NextA) was purchased from StarWise Pharmaceuticals, Madison, Wisconsin, USA. NextA was kept at a stock solution of 10 mg/mL and diluted with a buffer provided by the manufacturer to the concentration used for each particular experiment. Tubastatin A (S8049) was purchased from Selleckchem. MS275 (50-148-306) was purchased from Biotang Inc. The pan-HDACi LBH589 (50-148-338) was purchased from Biotang Inc. *In Vivo* MAb anti-mouse PD-1 (CD279) (BE0146) was manufactured and purchased from Bio X Cell (West Lebanon, New Hampshire, USA) and stored at 4 °C for use throughout the study.

### Flow cytometry

Mice were humanely euthanized, and tumor cells were immediately processed into a single cell suspension for analysis by flow cytometry. The first panel for flow cytometry analyzing the expression of immune cell surface markers was stained with phycoerythrin (PE) conjugated antibodies in a 96-well format. Antibodies were purchased from BD Biosciences (San Jose, California, USA), Biolegend (San Diego, California, USA) and eBioscience (San Diego, California, USA). Tumor sample cells were stained with anti-mouse CD274 (PD-L1)(BD Biosciences, 558091), anti-mouse CD 273(PD-L2)(Biolegend, 107205), anti-mouse CD276 (B7-H3) (Biolegend, 124507), anti-mouse B7-H4 (Biolegend, 139405), anti-mouse Galectin-9 (Biolegend, 137903), anti-mouse CD252 (OX40-L)(eBioscience, 12-5905-81), anti-mouse MHC I (H-2Kb)(eBioscience, 12-5958-80), anti-mouse MHC II (I-A/I-E) (eBioscience, 12-5321-81), anti-mouse DcTrail-R1 (TRAIL-R1)(Biolegend, 133804), anti-mouse CD262 (DR5)(TRAIL-R2)(eBioscience, 12-5883-82), anti-mouse CD80 (Biolegend, 104707), anti-mouse CD275(ICOS-L)(eBioscience, 12-5985-81), anti-mouse CD86 (Biolegend, 105007), anti-mouse CD137L (4-1BBL)(Biolegend, 107105), anti-mouse CD70 (Biolegend, 104605) and anti-mouse CD270 (HVEM) (Biolegend, 136303). After staining for 30 min at room temperature, cells were washed three times and then resuspended in FACS buffer.

The second panel for flow cytometry measured the activity and infiltration of Natural Killer (NK) and T-cells: PerCP/Cy5.5 anti-mouse CD3(T-cells) (Biolegend, 100218), Alexa Flour 488 anti-mouse CD4 (CD4+ T cells) (Biolegend, 100423), PE/Cy7 anti-mouse CD8a (CD8+ T cells) (Biolegend, 100766), APC/Fire 750 anti-mouse CD49b (NK cells) (Biolegend, 108922), Brilliant Violet 421 anti-mouse CD25 (T-cell activation) (Biolegend, 102034) and Brilliant Violet 785 anti-mouse CD45.2 (T-cell activation (Biolegend, 109839). The third flow cytometry panel measured the activity and infiltration of myeloid cells: Brilliant Violet 421 anti-mouse/human C11b (Macrophage)(Biolegend, 101236), APC anti-mouse CD80 (M1)(Biolegend, 104714), PE/Cy7 anti-mouse CD206 MMR (M2)(Biolegend, 141720), Brilliant Violet 630 anti-mouse CD11c (mDC)(Biolegend, 117339), APC/Fire 750 anti-mouse CD45.2 (MDSC) (Biolegend, 109832), PE anti-mouse CD123 (IL-3 receptor (IL-3R α))(Biolegend, 106003), Brilliant Violet 603 anti-mouse Ly-6G/Ly 6C (MDSC)(Biolegend, 108440), FITC anti-mouse H 2 (M2)(Biolegend, 125508), and Brilliant Violet 785 anti-mouse F4/80 (Macrophage)(Biolegend, 123141). After staining for 30 min at room temperature, cells were washed three times with 1X PBS. Samples were fixed with Life Technologies IC Fixation Buffer (FB001) from ThermoFisher Scientific (Waltham, Massachusetts, USA) according to the manufacturer’s protocol and then resuspended in FACS buffer.

The final flow cytometry panel analyzed the polarization of murine PEM cells. Collected PEM cells were stained with Alexa 488 anti-mouse F4/80 (Biolegend, 123120), PE/Cy7 anti-mouse CD206 (MMR) (Biolegend, 141720), APC anti-mouse CD80 (Biolegend, 104713) and PE MHC Class II (I-A/I-E) monoclonal antibody (eBioscience, 12-5321-81). The Fc block, anti-mouse CD16/32 (Biolegend, 101302), was used to prevent non-specific binding of the antibodies to samples. After staining for 30 min at room temperature, cells were washed three times and then resuspended in FACS buffer.

All flow cytometry panels used LIVE/DEAD® Fixable Aqua Dead Cell Stain (L34957) from ThermoFisher Scientific according to the manufacturer’s protocol to determine viability. At least 30,000 events were collected using a BD Celesta Cell Analyzer and analyzed using FlowJo software 10.4. The gating strategy used in the multicolor flow cytometry panels can be found in Supplementary Figs 7 and 8.

### Quantitative Real-time PCR

Total RNA was extracted from cells using the MiRNeasy Mini Kit according to the manufacturer’s instructions (Qiagen, Hilden, Germany, 217004). Samples were then processed immediately or stored at −80 °C. RNA was quantified using an ND-1000 NanoDrop Spectrophotometer (NanoDrop Technologies, Inc., Wilmington, Delaware). The 260/280 ratios were routinely over 1.9. Sample cDNA was produced using the iScript cDNA synthesis kit (Bio-Rad, 1708891). Target mRNA was quantified using MyIQ single color real-time PCR detection system from Bio-Rad (Bio-Rad, Hercules, CA) and iQ SYBR green Supermix (Bio-Rad, 1708882). Primers targeting PD-L1, PD-L2, Galactin-9, B7-H3, B7-H4, OX40-L, ICOS-L, IFNγ, IL-12, TGFβ and GAPDH for qRT-PCR were purchased from Invitrogen (Waltham, Massachusetts, USA) and the sequences are listed in Table 1. The primers for IL-2 (Quantitech, QT00112315) and IL-10 (Quantitech, QT00106169) were purchased from Qiagen (Hilden, Germany, 217004). Cycling conditions were used as per manufacturer’s instructions. Single product amplification was confirmed by melting curve analysis and primer efficiency was near 100% in all the experiments performed. Quantification is expressed in arbitrary units and target mRNA levels were normalized to GAPDH expression using the method described by Pfaffl *et al*.^57^ using Microsoft Excel Software (Microsoft, Redmond, WA).

**Table 1:** Primers used for qRT-PCR.

Primer Name	Sequence 5′ to 3′
PD-L1_Fwd	TAATCAGCTACGGTGGTGCG
PD-L1_Rev	CATGCTCAGAAGTGGCTGGA
PD_L2_Fwd	TGGAATGCTCACATGAAGGA
PD_L2_Rev	CCGGGATGAAAACATGAAGT
Galactin-9_Fwd	GCTTTACCCGTCCAAGTCCA
Galactin-9_Rev	CCTCCACAGCGAAGGTTGAT
B7-H3_Fwd	TGGGGCTCTCTGTCTGTCTT
B7-H3_Rev	TCTCCATCTCCATCCTGGTC
OX40-L_Fwd	CCAAAGACTCAGAGGAGCAGT
OX40-L_Rev	TCGCACTTGATGACAACCGA
ICOS-L_Fwd	GAGTTCACATGCCGGGTATT
ICOS-L_Rev	TCAGAGGTGCTGATGACAGG
IFNγ_Fwd	ACTGGCAAAAGGATGGTGA
IFNγ_Rev	GCTGATGGCCTGATTGTCTT
IL-12_Fwd	CCCATTCCTCGTCACGATCTC
IL-12_Rev	TCAGACTGGTTTGGGATAGGTTT
TGF_Beta_Fwd	ACCAACTATTGCTTCAGCTTCAGCTCCAC
TGF_Beta_Rev	GATCCACTTCCAACCCAGGTC
GAPDH_Fwd	ATGGCCTTCCGTGTTCCTAC
GAPDH_Rev	CAGATGCCTGCTTCACCAC
Granzyme B Fwd	CAGGAGAAGACCCAGCAAGTCA
GranzymeB Rev	CTCACAGCTCTAGTCCTCTTGG
Fas Ligand_Fwd	GAAGGAACTGGCAGAACTCCGT
Fas Ligand_Rev	GCCACACTCCTCGGCTCTTTTT
TRAIL_Fwd	GGAAGACCTCAGAAAGTGGCAG
TRAIL_Rev	TTTCCGAGAGGACTCCCAGGAT

### Immunoblotting

The cells were lysed in RIPA buffer (Pierce, 89900) with 1X protease and phosphatase inhibitor (Pierce, A32961). Lysates were sonicated in a Bioruptor™ (Diagenode, Denville, NJ, USA) on ice for 8 minutes (8 cycles of 30 s on, 30 s off). Protein concentration was determined using a Pierce BCA Protein Assay Kit (Thermo Fisher Scientific, 23225) according to the manufacturer’s protocol. Samples were mixed with NuPAGE LDS 4x loading gel (NP0007) and NuPAGE 10x reducing agent (NP0009), and boiled at 95 °C. Next, samples were loaded onto 4–20% (BioRad, 4561093) or 10% gels (BioRad, 4561033) and transferred to LF PVDF (BioRad, 170–4274). Membranes were blocked with LI-COR Biosciences (Lincoln, Nebraska, USA) Odyssey Blocking Buffer (927–40100). Bands were detected using Azure Biosystems (Dublin, California, USA) Imaging System c600. The antibodies used for immunoblotting included: PD-L1 (ProSci, 4059), PD-L2 (ProSci, 4063), CD70 (Abcam, ab175389), B7-H3 (ThermoFisher Scientific, PA551098), B7-H4 (Abbiotec, 250473), Galectin-9 (Abcam, ab9630), ICOS-L (Abcam, ab138354), alpha-Tubulin (Cell Signaling, 3873) and acetyl-alpha Tubulin (Cell Signaling, 3971).

### Statistical Analysis and reproducibility

All experiments were done in triplicate unless otherwise noted. Analysis completed using unpaired t-tests and ANOVA with significance at p < 0.05 and the Kaplan-Meier survival curves were conducted using GraphPad Prism 7. All analyses of cell viability, apoptosis and cytotoxicity were conducted using Microsoft Excel Software (Microsoft, Redmond, WA).

## Supplementary information

Supplementary Dataset 1
