# Author Correction: Selective HDAC6 inhibitors improve anti-PD-1 immune checkpoint blockade therapy by decreasing the anti-inflammatory phenotype of macrophages and down-regulation of immunosuppressive proteins in tumor cells

**DOI:** 10.1038/s41598-019-51403-6

**Published:** 2019-10-10

**Authors:** Tessa Knox, Eva Sahakian, Debarati Banik, Melissa Hadley, Erica Palmer, Satish Noonepalle, Jennifer Kim, John Powers, Maria Gracia-Hernandez, Vasco Oliveira, Fengdong Cheng, Jie Chen, Cyril Barinka, Javier Pinilla-Ibarz, Norman H. Lee, Alan Kozikowski, Alejandro Villagra

**Affiliations:** 1 The George Washington University, Washington, DC USA; 2 H. Lee Moffitt, Tampa, FL USA; 3 Hospital Episcopal San Lucas, Ponce, PR USA; 4 Institute of Biotechnology of the Czech Academy of Sciences, BIOCEV, Vestec, Czech Republic; 5 StarWise LLC, Madison, WI USA

Correction to: *Scientific Reports* 10.1038/s41598-019-42237-3, published online 16 April 2019

The Acknowledgements section of this Article is incomplete.

“Funded by NIH R21 CA184612-01 and Melanoma Research Foundation CDA Grant Award (A.V.). NIH R01 CA204806 (N.L.). We would like to acknowledge the important technical contributions and advice of Kimberlyn Acklin, MS, SCYM(ASCP), at The George Washington University Flow Cytometry Core Facility and Bethany Rentz, RVT, at The George Washington University Office of Animal Research.”

should read:

“Funded by NIH R21 CA184612-01 and Melanoma Research Foundation CDA Grant Award (A.V.), NIH R01 CA204806 (N.L.), Czech Science Foundation (No 15-19640S), the CAS (RVO: 86652036) and the project „BIOCEV” (CZ.1.05/1.1.00/02.0109) from the ERDF (C.B.). We would like to acknowledge the important technical contributions and the advice of Kimberlyn Acklin, MS, SCYM(ASCP), at The George Washington University Flow Cytometry Core Facility and Bethany Rentz, RVT, at The George Washington University Office of Animal Research.”

