# Supplementary material for: Selective HDAC6 inhibitors improve anti-PD-1 immune checkpoint blockade therapy by decreasing the anti-inflammatory phenotype of macrophages and down-regulation of immunosuppressive proteins in tumor cells
Source: Sci Rep. 2019 Apr 16;9:6136. doi: 10.1038/s41598-019-42237-3 (PMC6467894; doi:10.1038/s41598-019-42237-3)

Selective HDAC6 inhibitors improve anti-PD-1 immune checkpoint blockade therapy by decreasing the anti-inflammatory phenotype of macrophages and down-regulation of immunosuppressive proteins in tumor cells.

Tessa Knox[1#], Eva Sahakian[2#], Debarati Banik[1], Melissa Hadley[1], Erica Palmer [1], Satish Noonepalle[1], Jennifer Kim[1], John Powers[2], Maria Gracia-Hernandez[1], Vasco Oliveira[3], Fengdong Cheng[1], Jie Chen[1], Cyril Barinka[4], Javier Pinilla-Ibarz[2], Norman H. Lee[1], Alan Kozikowski[5] , and Alejandro Villagra[1*]

Supplementary Figure 1. **HDAC6 selective inhibitors modulate immune-regulatory mediators without inducing cytotoxicity.**

(A) NextA (5μM) and untreated murine SM1 melanoma cells were incubated with IFNγ for 24hrs, and the expression of PD-L1, PD-L2, and tubulin were evaluated by immunoblot. (B) Similarly, SM1 cells were treated with NextA (5μM) and the expression of HDAC6, ac-tubulin, galectin-9, ICOSL, CD70, B7-H3, B7-H4, and tubulin evaluated by immunoblot. (C) Lung tissue from HDAC6 knock-out or wild type mice was screened for the expression of the proteins noted in the figure.

Supplementary Figure 2. **HDAC6i and anti-PD-1 blocking antibody decrease tumor growth in a dose dependent manner.**

(A) C57BL/6 mice were treated by intraperitoneal injection 5 times a week with a vehicle control or NextA at the various doses indicated in order to determine the optimal treatment dose. (B) C57BL/6 Mice were treated by intraperitoneal injection 3 times a week with manti-PD1 mAb at the various doses indicated in order to determine the optimal treatment dose. (C) Mice were given intraperitoneal injections 5 times a week with Isotype IgG2 at 15 mg/kg or a vehicle control and tumor growth monitored for 21 days.

Supplementary Figure 3. Inhibition of HDAC6 improves immune check-point blockade.

(A) C57BL/6 mice were subcutaneously injected with $1x10^6$ B16-F10 murine melanoma tumor cells. Mice were treated with a vehicle control, 15 mg/kg anti-PD-1, 25 mg/kg NextA, or a combination of both agents. (B) Kaplan-Meier survival plot of the previous study. (C) Individual group plots representation for the previous study.

Supplementary Figure 4. **NextA modulates the expression of immune-regulatory mediators.**

C57BL/6 mice were subcutaneously injected with $1x10^6$ SM1 murine melanoma tumor cells. Mice were treated with a vehicle control, 15 mg/kg anti-PD-1, 25 mg/kg NextA, or a combination of both agents for 30 days. The NextA pre-treatment condition noted in the figure started 10 days before other conditions. Tumors were collected at the study end-point and the expression of PD-L1, PD-L2, B7-H3, OX40L, galectin-9 and ICOSL was evaluated by qRT-PCR (A-F). Similarly, the presence of CD80, CD86, CD70, HVEM, Gal9, ICOSL, B7-H3, and B7-H4 was evaluated by flow cytometry (G-N).

Supplementary Figure 5. **HDAC6 knock-down diminish tumor growth.**

(A) Individual group plots representation for the in vivo study depicted in figure 4E. C57BL/6 mice were subcutaneously injected with $1x10^6$ SM1 murine melanoma cells lacking HDAC6 (HDAC6KD) or control cells (non-target). Tumor nodules were isolated to evaluate CD8+ and CD4+ infiltration (B), and the presence of PD-1, LAG-3 and TIM3 (C). HDAC6-/- mice were subcutaneously injected with $1x10^6$ SM1 murine melanoma tumor cells. Tumor nodules were isolated to evaluate CD8+ and CD4+ infiltration (D), and the presence of PD-1, LAG-3 and TIM3 (E).

Supplementary Figure 6. Macrophage depletion impairs tumor growth.

1x106 SM1 cells were injected subcutaneously in the flanks of C57/Bl6 mice. When tumors became palpable, mice were injected intratumorally with Clodrosomes or Encapsomes (vehicle) for two weeks. The composition of M1, M2, DC, and T cells was evaluated from end-point tumors (F), and tumor growth measured during the two week of treatment (G).

Supplementary Figure 7 and 8. Gating strategy for the tumor infiltrating lymphocyte flow cytometry panels.

Supplementary Figure 9 and 10. Representative cell counts for each condition shown in Figures 6B-M.

Supplementary Figure 1

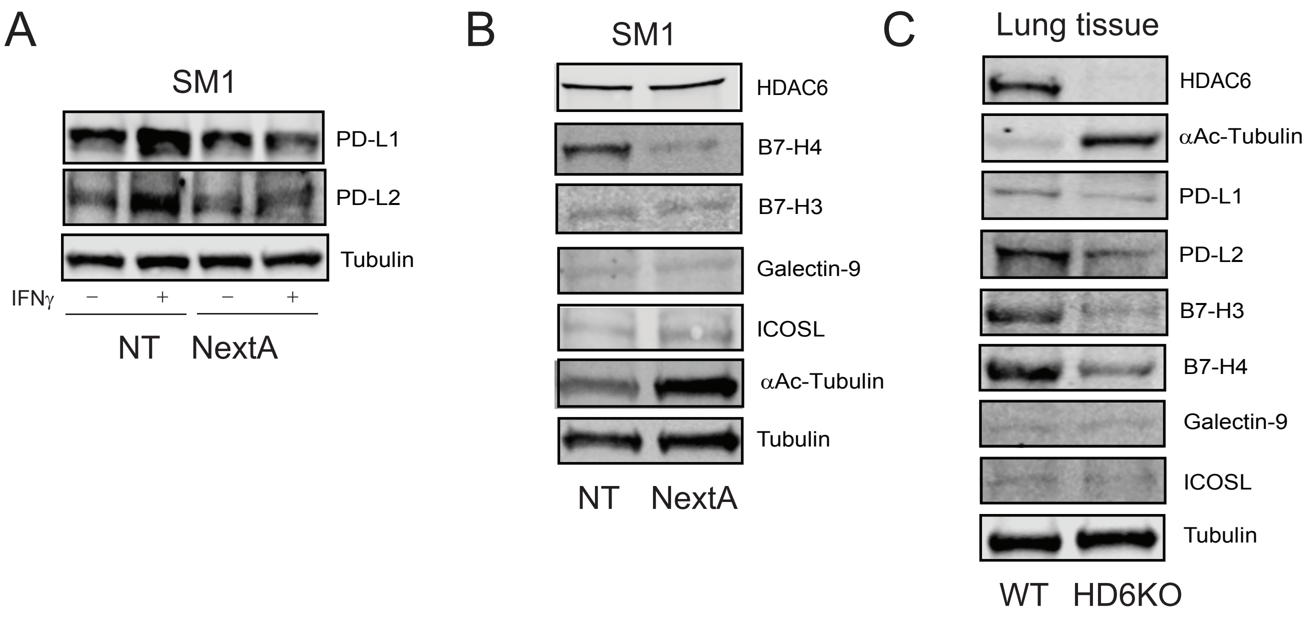

Supplementary Figure 2

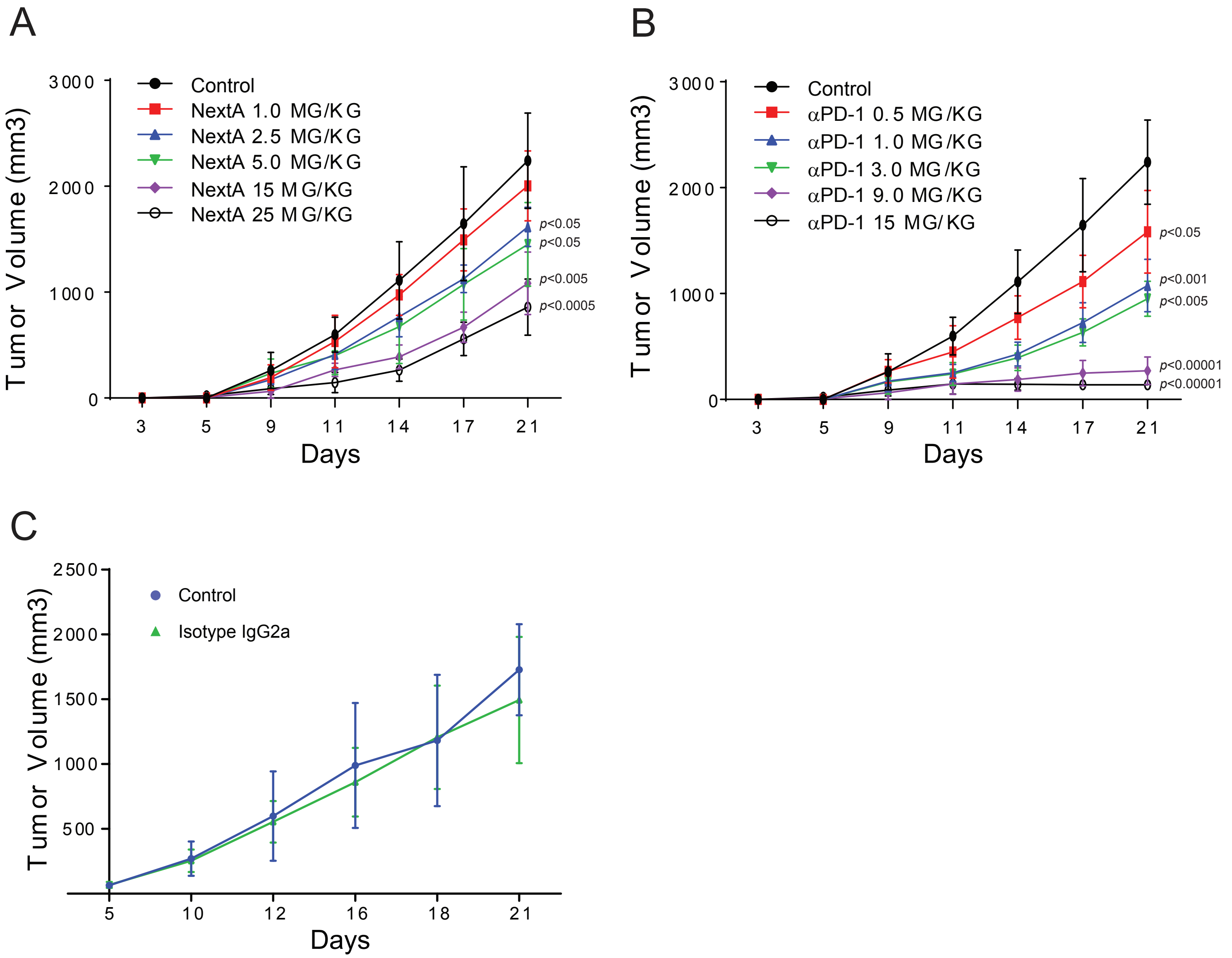

Supplementary Figure 3

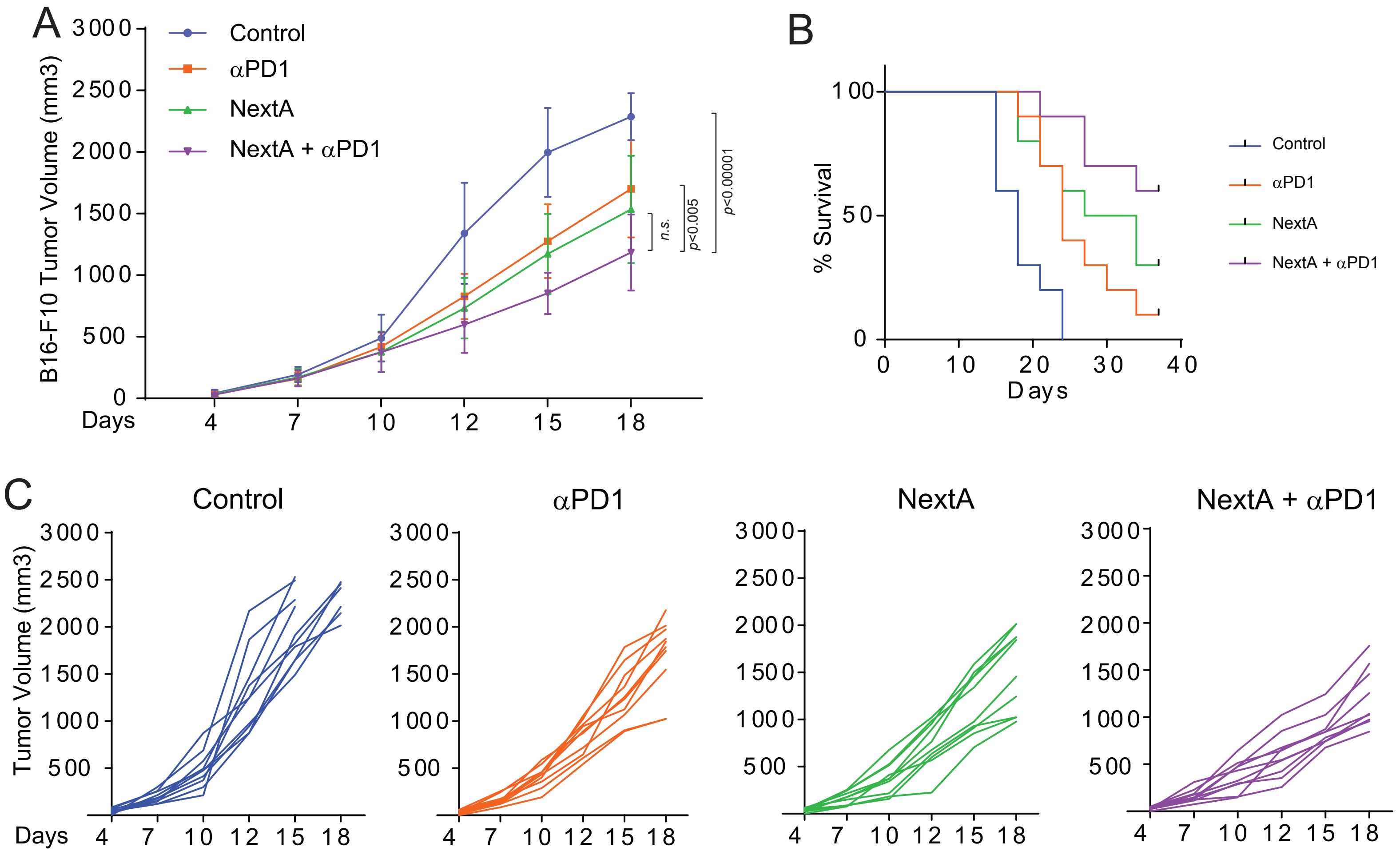

Supplementary Figure 4

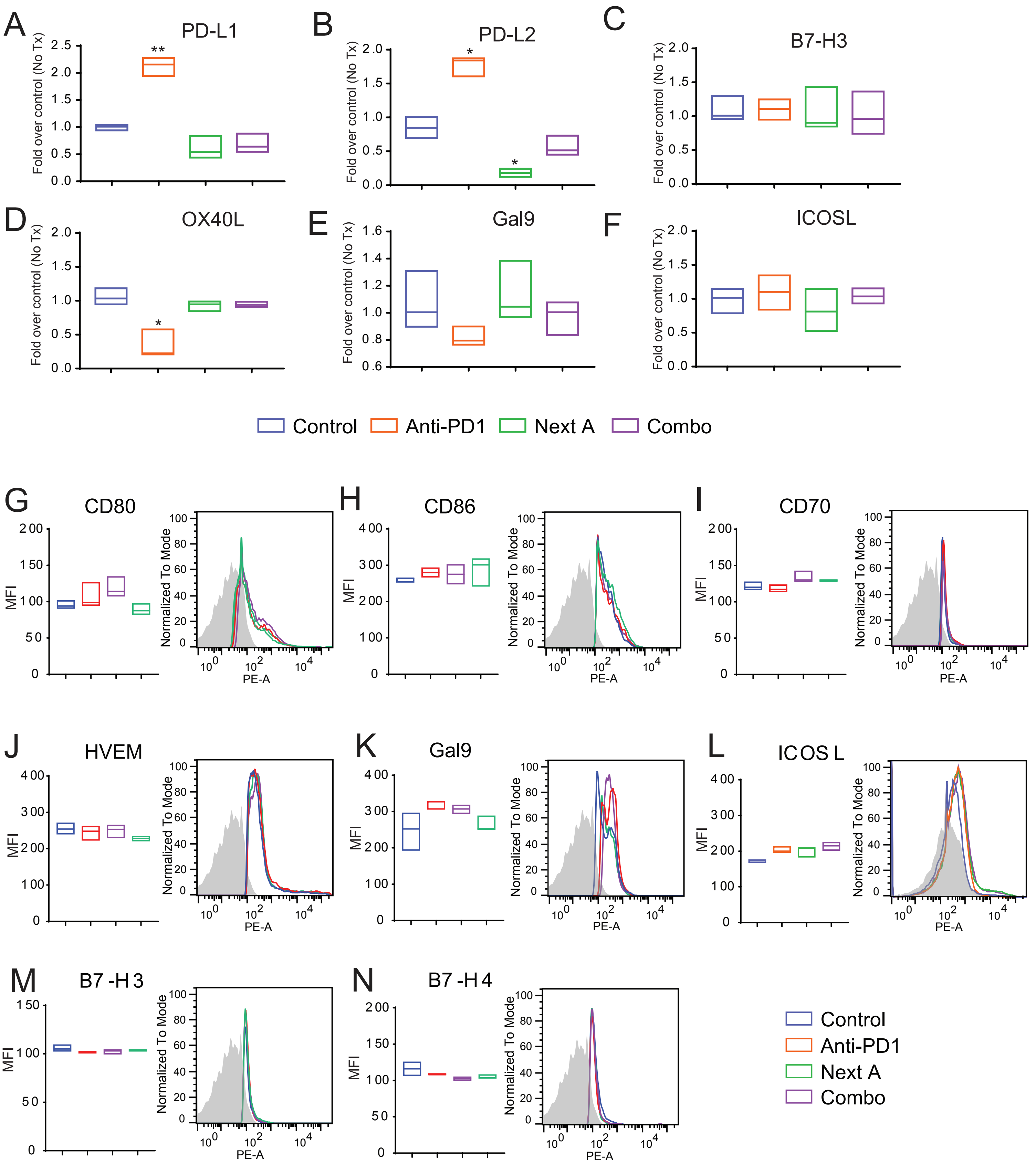

Supplementary Figure 5

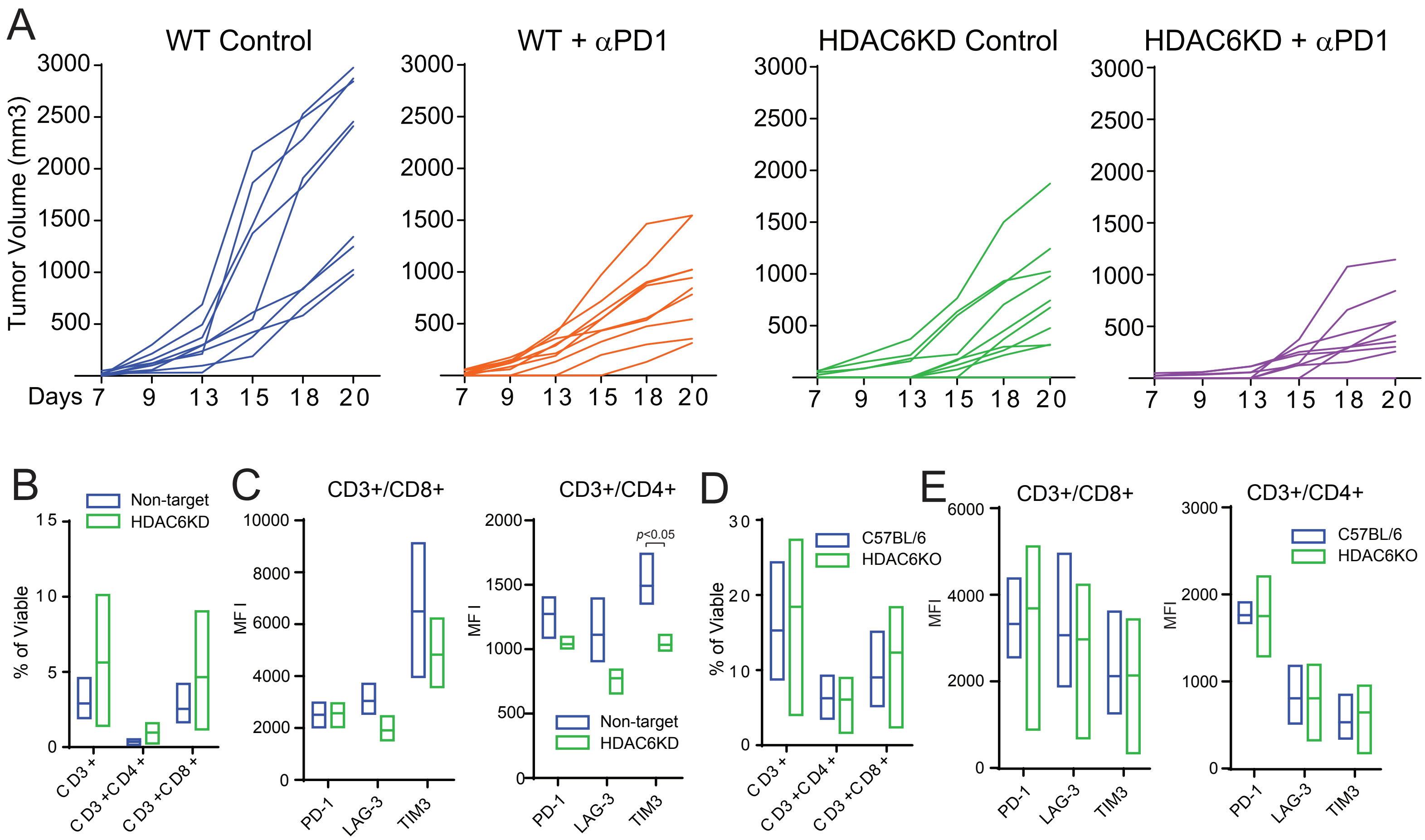

Supplementary Figure 6

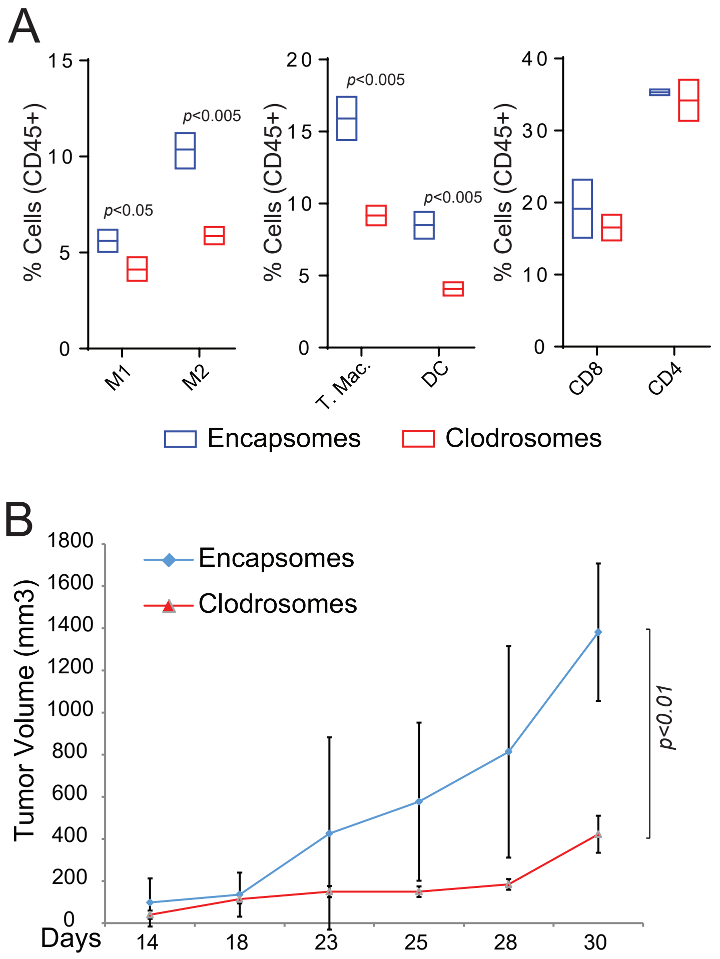

Supplementary Figure 7

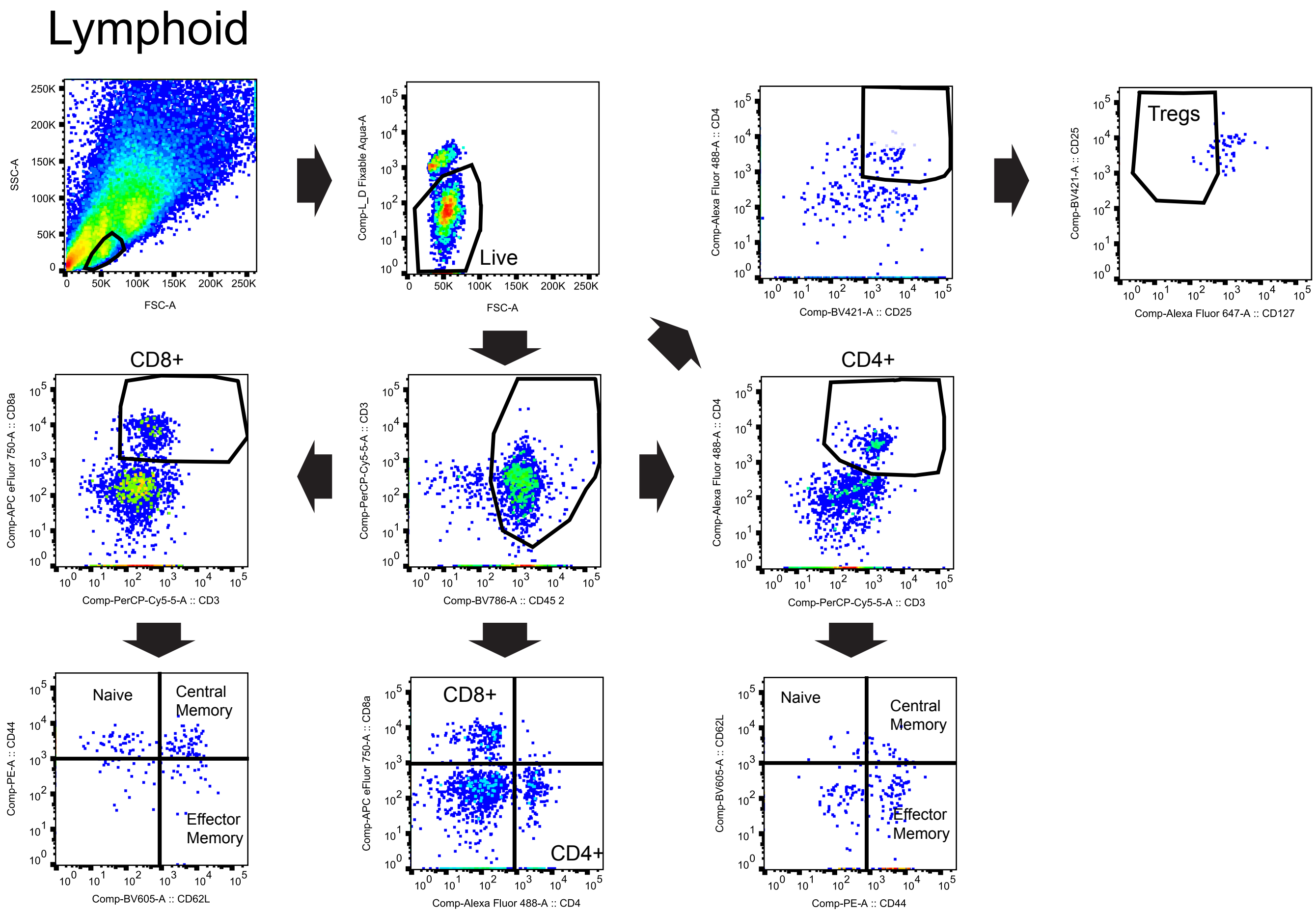

Supplementary Figure 8

NK

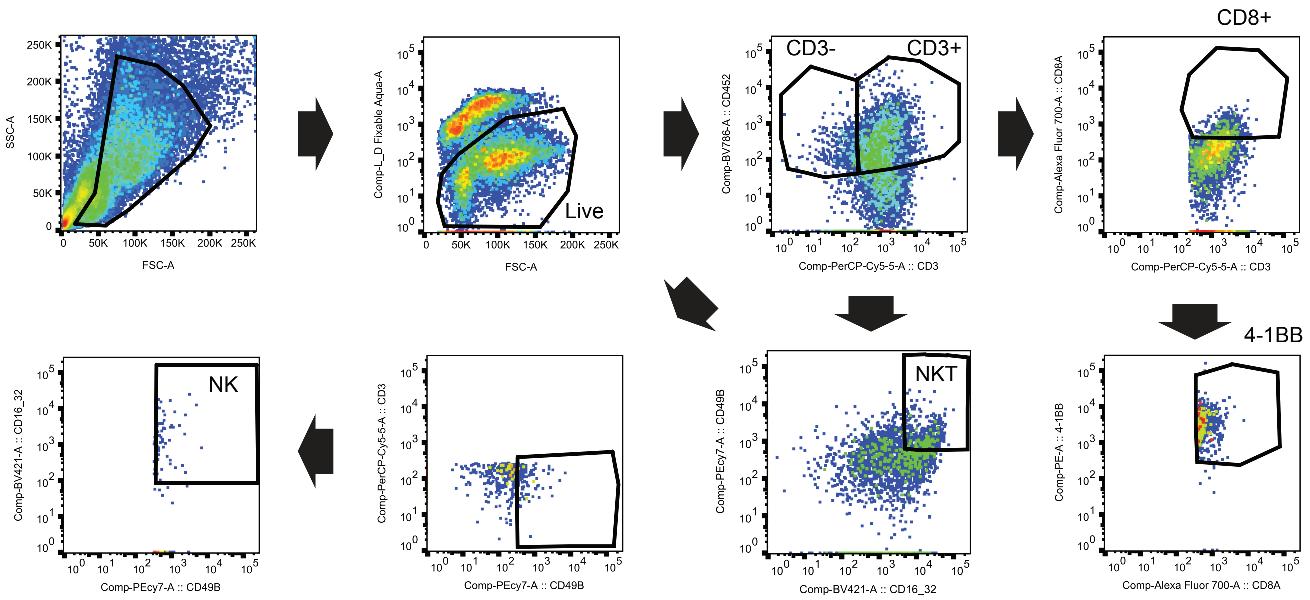

Myeloid

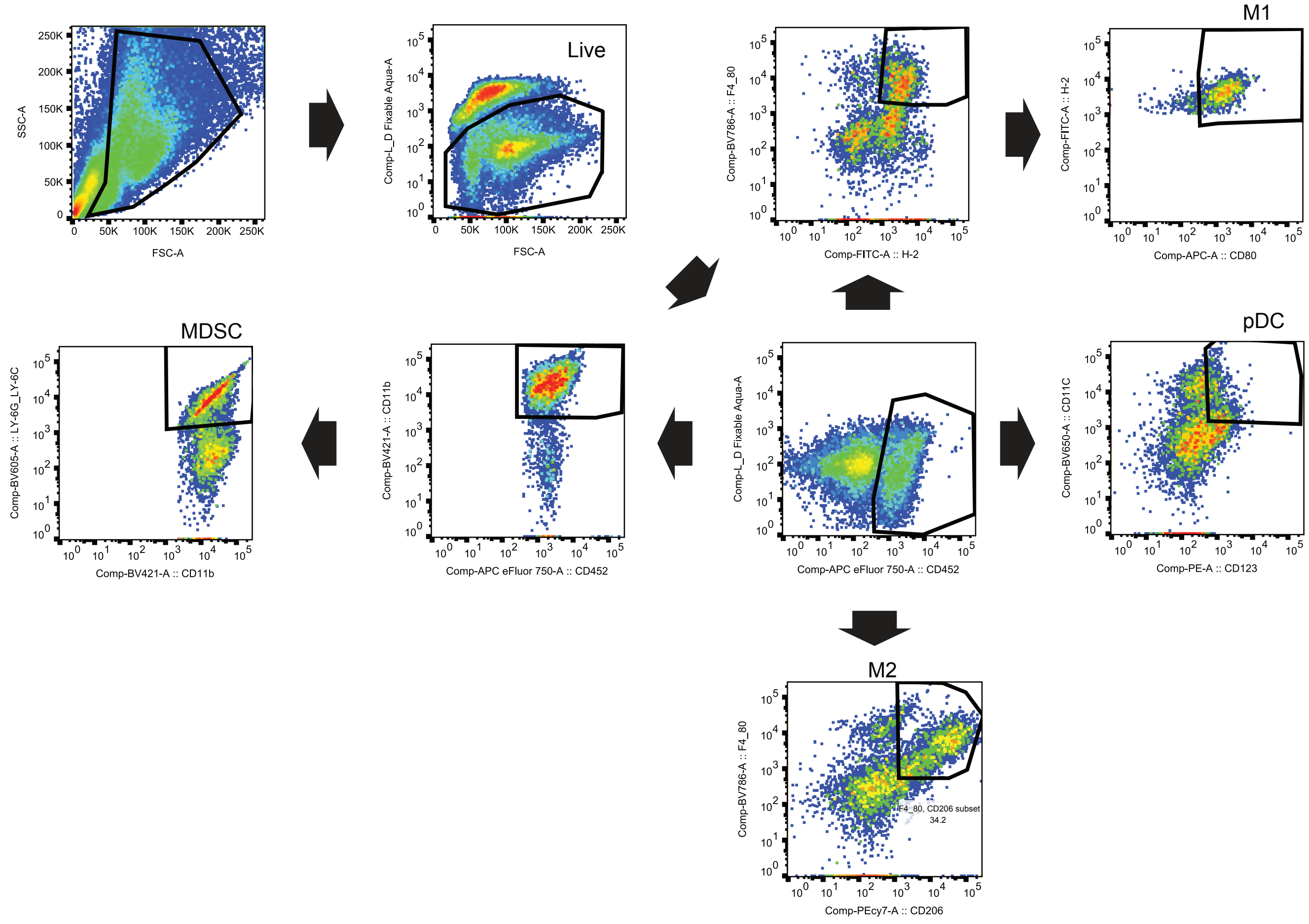

Supplementary Figure 9

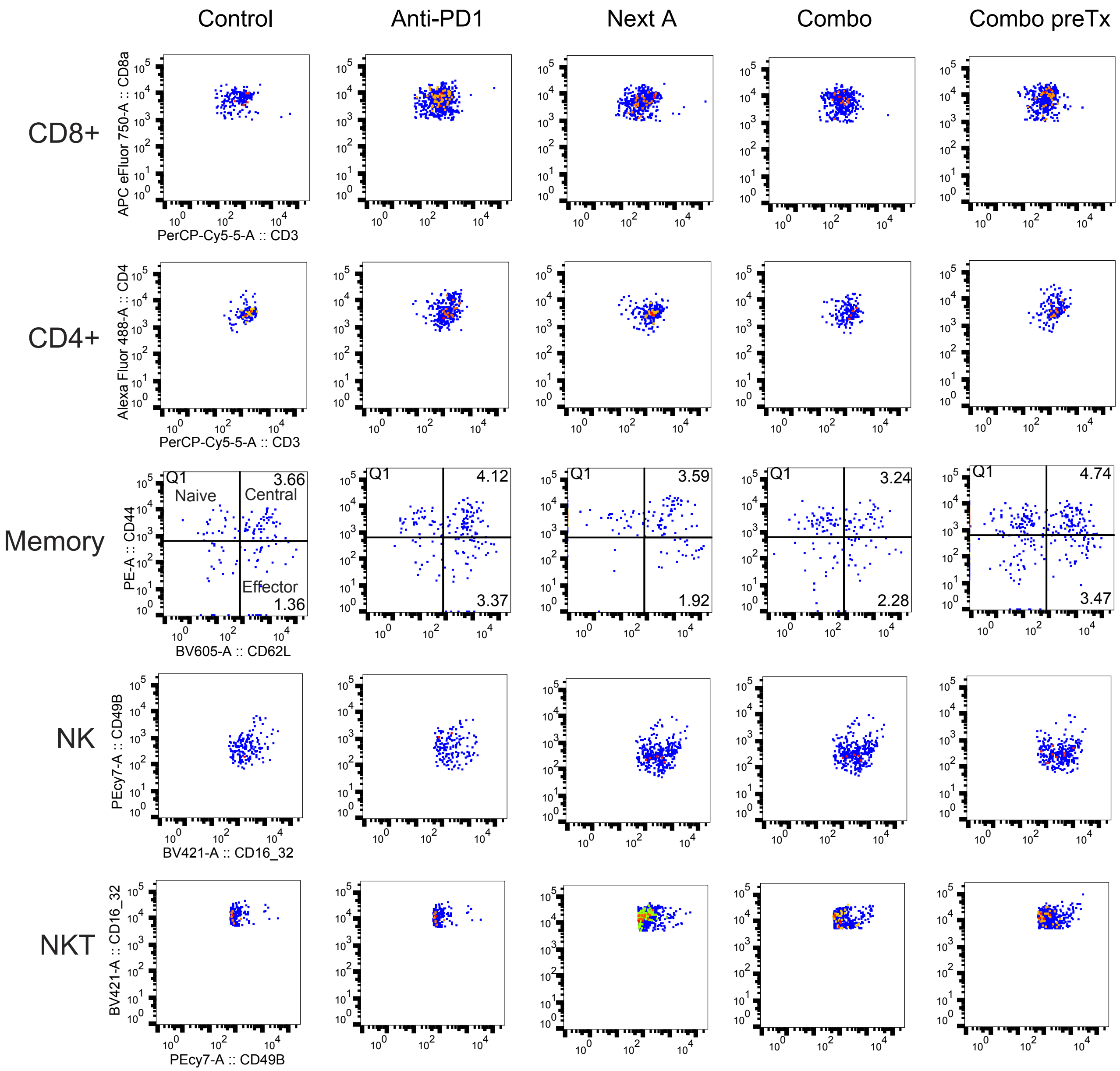

Supplementary Figure 10

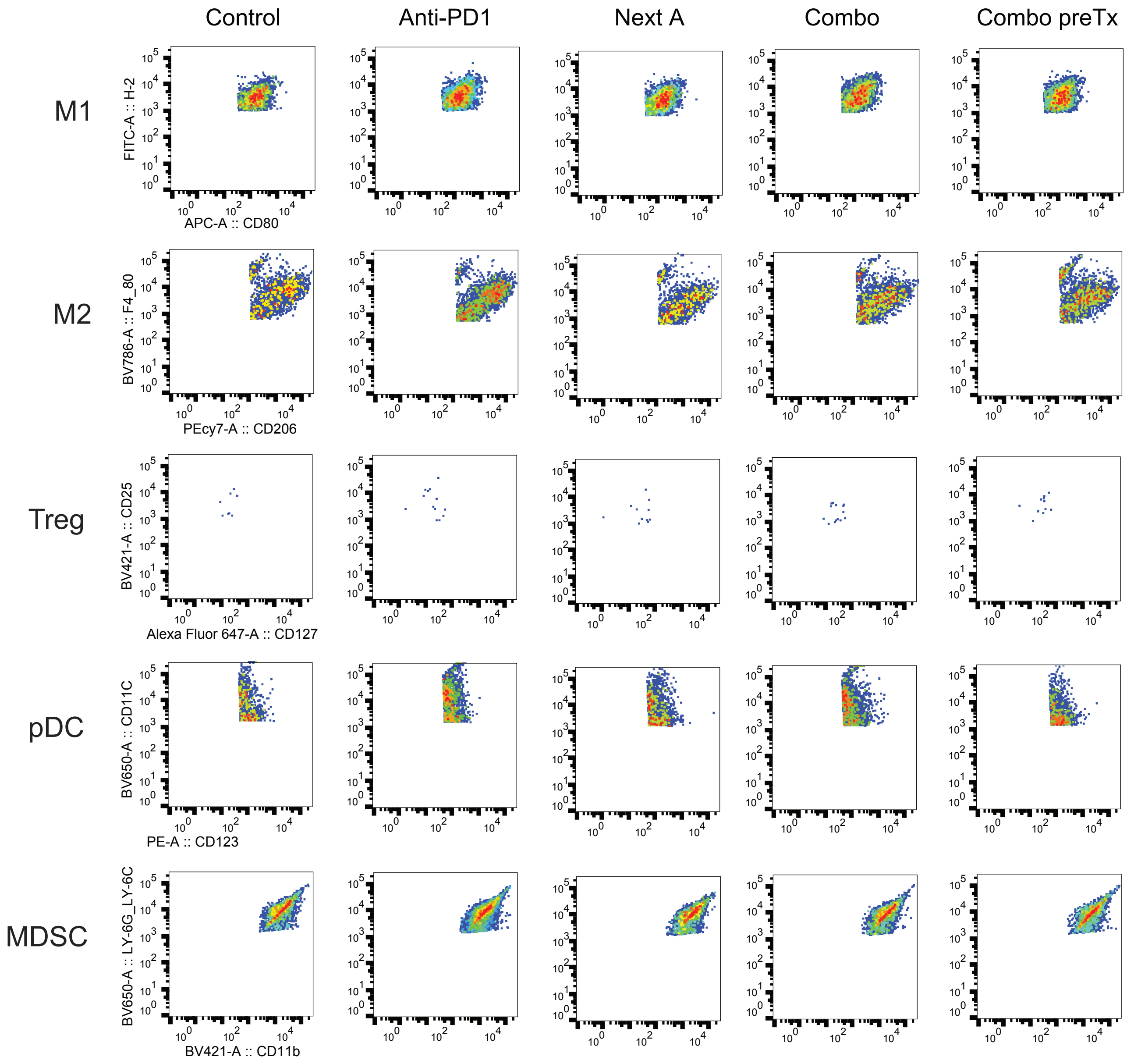

Full images of western blots in Figure 1.C

1. Control 1
2. Control 2
3. Anti-PD1 antibody therapy 1
4. Anti-PD1 antibody therapy 2

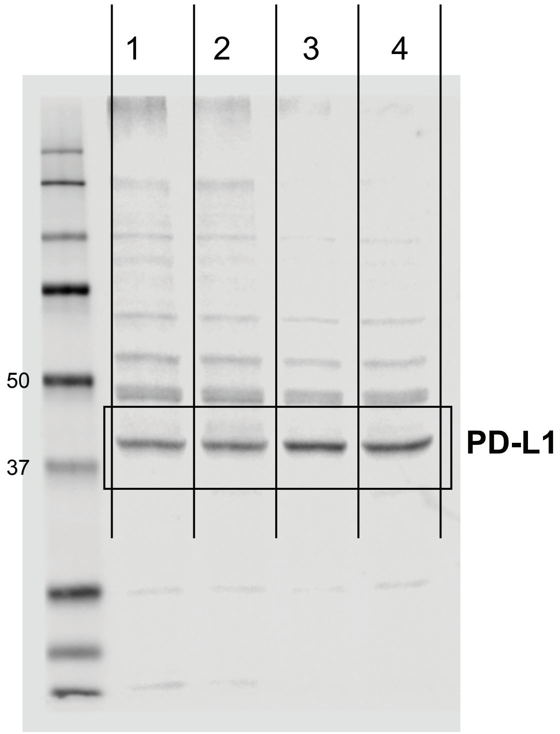

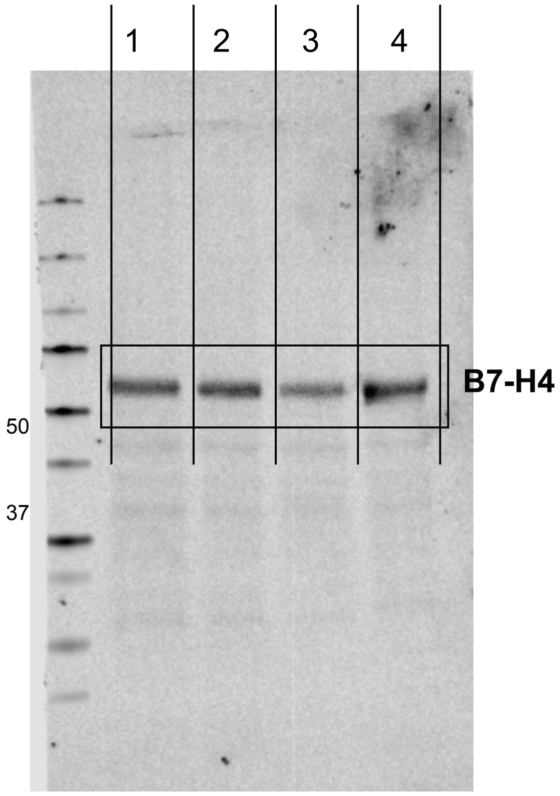

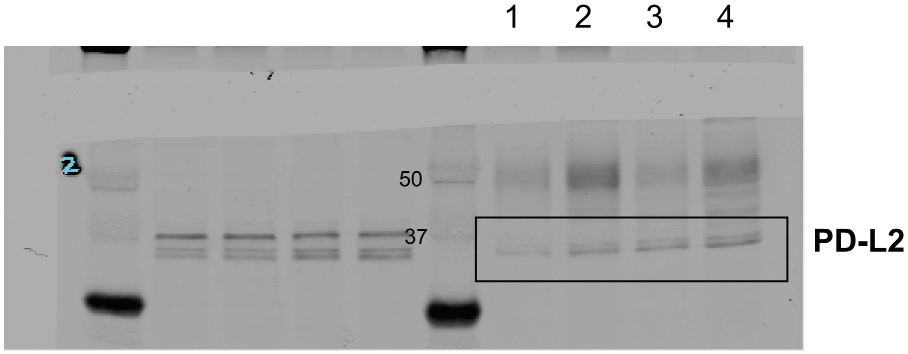

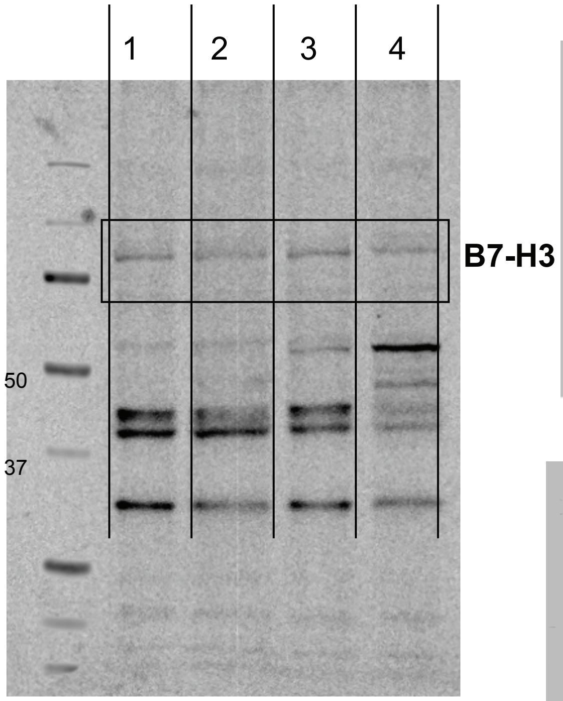

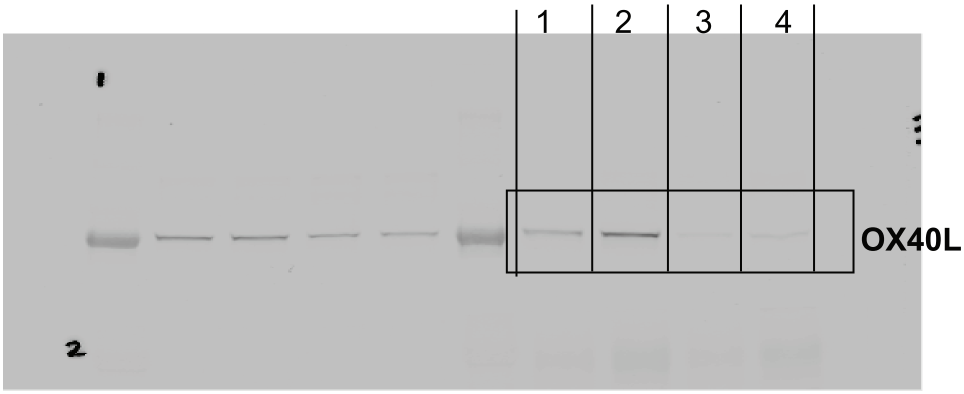

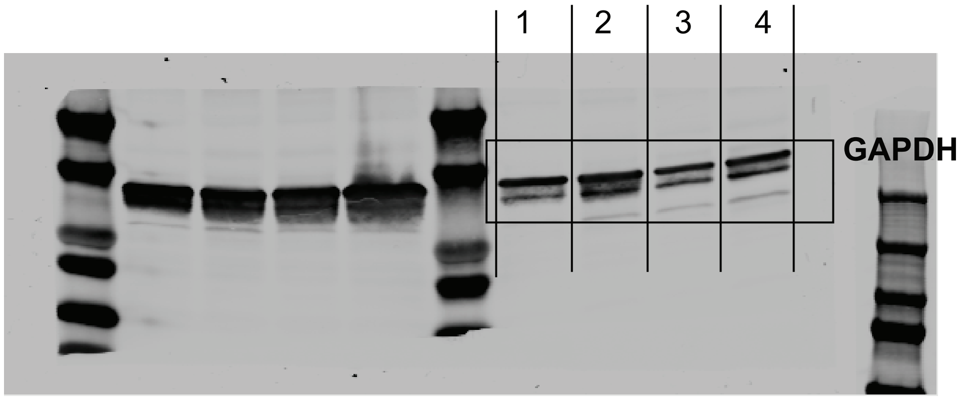

Full images of western blots in Figure 2.I

1. Control
2. Anti-PD1 antibody therapy
3. Nexturastat A therapy
4. Combination therapy

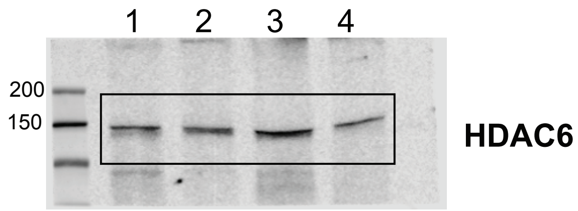

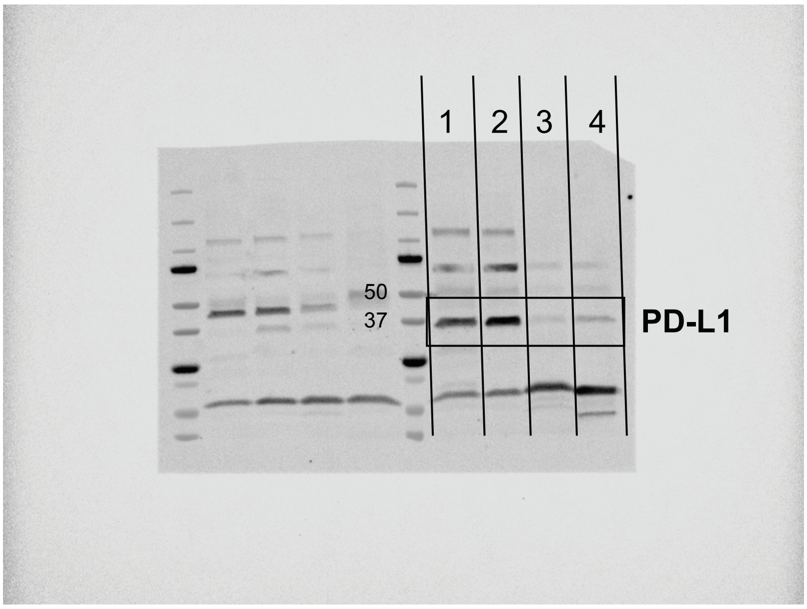

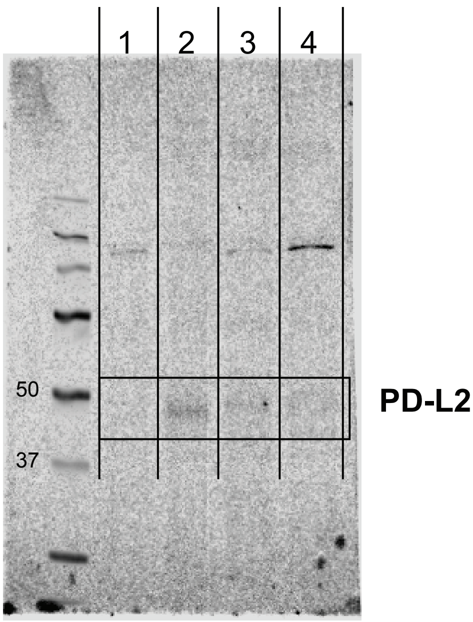

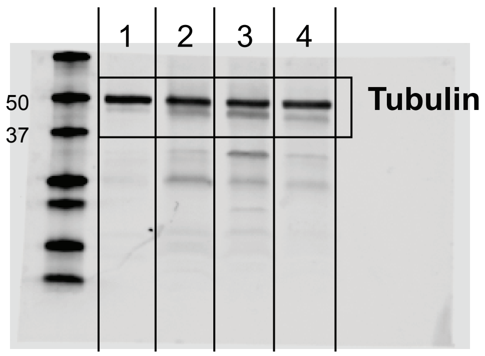

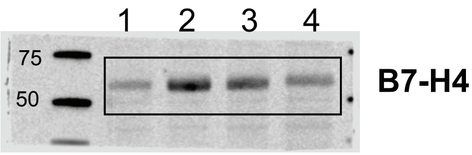

Full images of western blots in Supplementary Figure 1

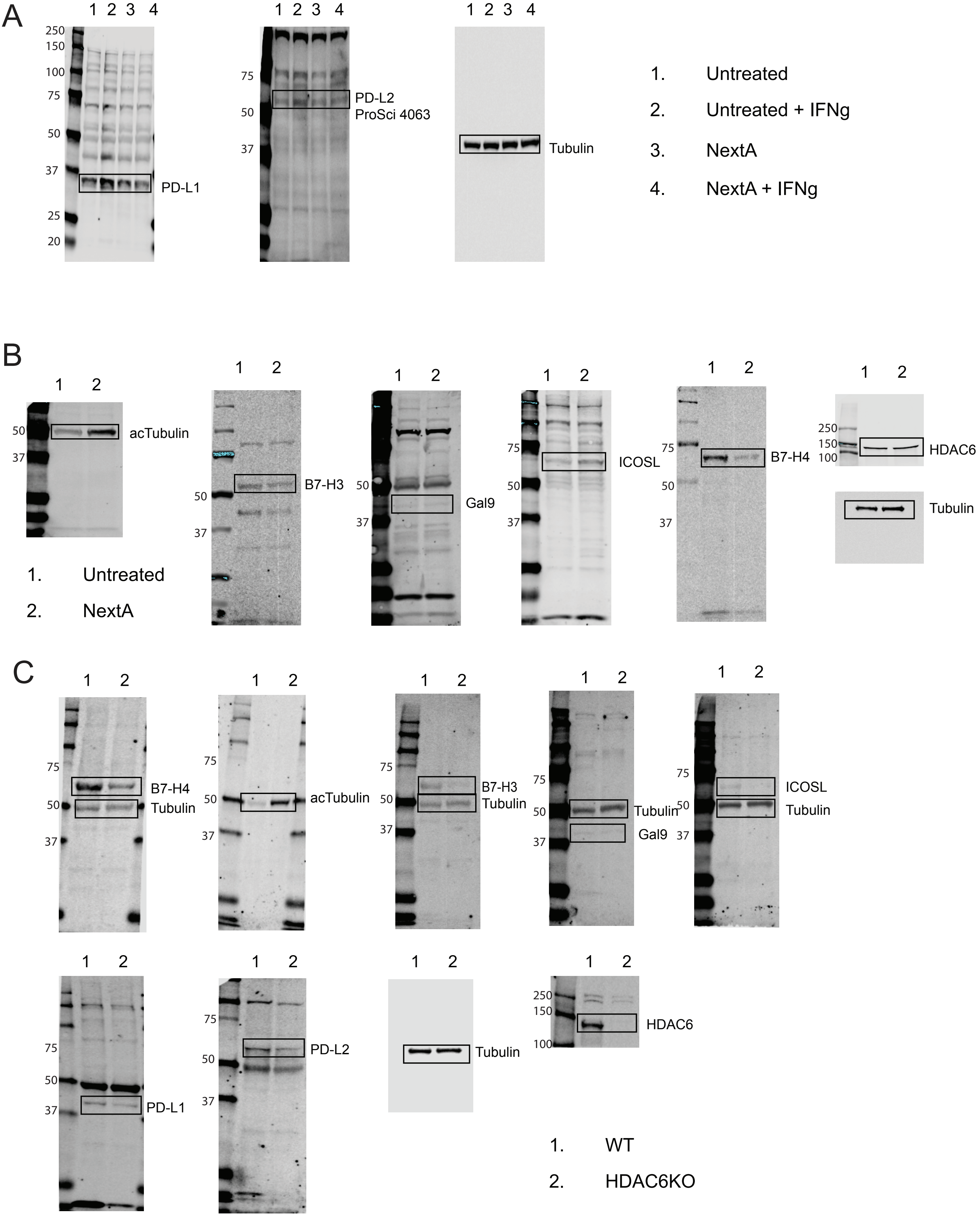

Supplement: Supplementary file 1 — Supplementary Dataset 1 [file 41598_2019_42237_MOESM1_ESM.pdf]
